# SPIKE: secure and private investigation of the kidney exchange problem

**DOI:** 10.1186/s12911-022-01994-4

**Published:** 2022-09-22

**Authors:** Timm Birka, Kay Hamacher, Tobias Kussel, Helen Möllering, Thomas Schneider

**Affiliations:** 1 ENCRYPTO, Technical University of Darmstadt, Darmstadt, Germany; 2 Computational Biology and Simulation group, Technical University of Darmstadt, Darmstadt, Germany

**Keywords:** Kidney-exchange, Privacy, Secure multi-party computation (MPC)

## Abstract

**Background:**

The kidney exchange problem (KEP) addresses the matching of patients in need for a replacement organ with compatible living donors. Ideally many medical institutions should participate in a matching program to increase the chance for successful matches. However, to fulfill legal requirements current systems use complicated policy-based data protection mechanisms that effectively exclude smaller medical facilities to participate. Employing secure multi-party computation (MPC) techniques provides a technical way to satisfy data protection requirements for highly sensitive personal health information while simultaneously reducing the regulatory burdens.

**Results:**

We have designed, implemented, and benchmarked SPIKE, a secure MPC-based privacy-preserving KEP protocol which computes a locally optimal solution by finding matching donor–recipient pairs in a graph structure. SPIKE matches 40 pairs in cycles of length 2 in less than 4 min and outperforms the previous state-of-the-art protocol by a factor of $400\times$ in runtime while providing medically more robust solutions.

**Conclusions:**

We show how to solve the KEP in a robust and privacy-preserving manner achieving significantly more practical performance than the current state-of-the-art (Breuer et al., WPES’20 and CODASPY’22). The usage of MPC techniques fulfills many data protection requirements on a technical level, allowing smaller health care providers to directly participate in a kidney exchange with reduced legal processes. As sensitive data are not leaving the institutions’ network boundaries, the patient data underlie a higher level of protection than in the currently employed (centralized) systems. Furthermore, due to reduced legal barriers, the proposed decentralized system might be simpler to implement in a transnational, intereuropean setting with mixed (national) data protecion laws.

**Supplementary Information:**

The online version contains supplementary material available at 10.1186/s12911-022-01994-4.

## Introduction

Around 7% of U.S. adults are affected by chronic kidney disease [1]. With the increasing age of the population in most countries, end-stage renal disease constitutes a rapidly increasing challenge for health care systems [2]. Humans are able to live a normal life with at least one functioning kidney [3]. However, when both kidneys of a person are malfunctioning, this person requires kidney replacement therapy to survive, i.e., either dialysis or the donation of a functioning kidney.

Transplantations of deceased donor organs unfortunately imply long waiting times, as transplant waiting lists grow, given that the number of donations significantly exceed supply [4]. The other option is to find a living person that is willing to donate one of their kidneys. In general, living donor donations result in shorter waiting times and tend to have better long term outcomes compared to deceased donor donations [5]. Unfortunately, finding a willing, living donor does not guarantee (medical) compatibility with the recipient. Hence, the living donor exchange system was introduced in 1991 [6], which allows recipients with incompatible living donors, in the following referenced as *pairs*, to exchange their donors, such that ideally each recipient can receive a compatible kidney donation. In most European kidney exchange programs the kidney transplantations of an exchange are executed simultaneously. Simultaneous operations limit the length of exchange cycles due to scarcity of medical staff. Additionally, exchanges of an exchange cycle that were initially deemed compatible in SPIKE can still be deemed incompatible after the required further assessment done by medical professionals. In case of longer exchange cycles, this leads to more pairs not receiving a kidney due to the failing of the whole exchange cycle. For these reasons, many European countries with kidney exchange programs limit the length of cycles to $L=3$ or even $L=2$, ensuring a practical feasibility [7]. In order for SPIKE to be applicable in European kidney exchange programs^1^, we decided to limit the maximum length of cycles to $L=3$.

In this work, we consider a scenario, in which several pairs exchange their donors in a cyclic fashion, so that each donating pair receives a compatible kidney. These cycles are called *exchange cycles* [7].

**Fig. 1:**
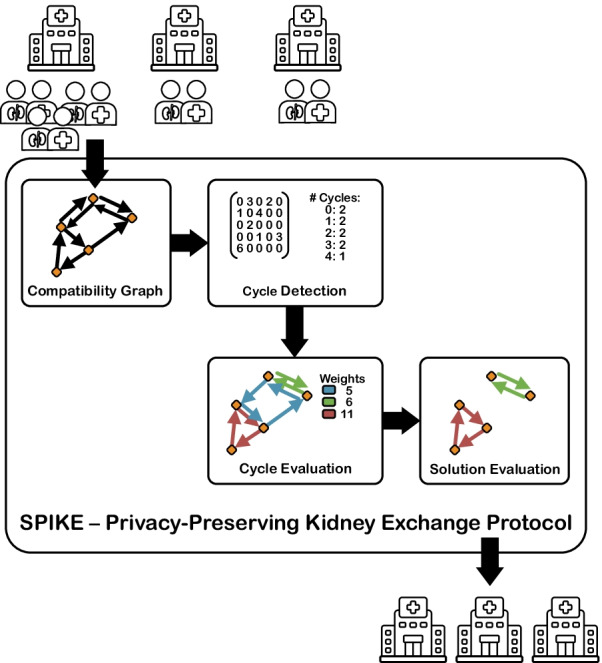
Overview of our privacy-preserving kidney exchange protocol SPIKE. The best set of exchange cycles are calculated, while the patients’ data remain strictly private

As a first step for finding possible exchange cycles, we have to evaluate the donors’ and recipients’ medical data to determine compatibility between pairs. Afterwards, we have to identify possible exchange cycles. This problem is known as the kidney exchange problem (KEP) [7] and can be described as finding cycles in a directed graph, where each vertex represents a pair and a directed edge describes the compatibility between two pairs. A schematic view of the protocol can be seen in Fig. 1.

The process requires the analysis of highly sensitive medical health data, which makes it crucial that no information is leaked accidentally or to unauthorized personnel. Thus, the KEP requires the implementation of strong privacy-preserving solutions, where the plaintext health information remains locally at each medical institution and the analysis is only run on “encrypted” data, which is leaking no sensitive data beyond the output: an exchange cycle with high transplantation success likelihood.^2^ Note that such a distributed solution also enhances security against data breaches, as having to attack multiple parties is significantly harder than a single target. Similarly, it also simplifies the compliance with regulatory requirements potentially complicating or even prohibiting data sharing among facilities.

### Contributions and outline

In this work, we provide the following contributions:

- *Efficient Privacy-Preserving Kidney Exchange protocol:* We design and implement SPIKE, a distributed, privacy-preserving protocol for solving the kidney exchange problem in the semi-honest security model. In contrast to the current state-of-the-art [9, 10], SPIKE improves efficiency as well as the medical compatibility matching by considering additional factors, namely, age, sex, human leukocyte antigens, and weight, that significantly affect compatibility between potential donors and recipients and is, thus, more robust than previous solutions by reducing the risk of failing procedures.
- *Comprehensive Empirical Evaluation:* We implement and extensively benchmark SPIKE and show that it significantly improves runtimes and communication costs compared to the state-of-the-art. We achieve about ${30000}\times$ speedup over [9] and ${400}\times$ over [10] thanks to our carefully optimized hybrid secure multi-party computation (MPC) protocols. Further, we provide additional (micro-) benchmarks and network settings to further demonstrate scalability and practicality of SPIKE.
- *Open-source Implementation:* SPIKE is available under the GNU LGPL v3 license^3^ here: https://encrypto.de/code/PPKE.

## Related work

In this section, we summarize the related work on the Kidney Exchange Problem (KEP) with and without considering data privacy.

### Robust KEP solutions

One major issue in kidney exchange programs is the potential cancellation of transplantations *after* having already determined exchange cycles of compatible donors and recipients. Reasons for such cancellations are manifold, e.g., a donor withdraws his consent, as his non-compatible relative has already received a kidney via the waiting list from a deceased donor in the meantime [11]. These issues call for *robust* solutions to the KEP, i.e., flexibility for recipient/donor dropouts and including as much as possible medical factors that can be algorithmically evaluated.

Carvalho et al. [12] propose three policies that are able to cope with dropouts within an kidney exchange cycle. One takes the costs (or missed gains) of planned transplants that do not proceed into account to find a solution with high probability of being successfully executed. The other two policies investigate strategies for recovering exchange cycles after dropouts. The plaintext algorithms in [12] are computationally expensive and, thus, cannot be trivially realized as secure computation protocols.

Ashby et al. [13] introduce a calculator for determining compatibility in kidney exchange, which they use to evaluate the importance of various medical factors, such as age, sex, obesity, weight, height, human leukocyte antigen (HLA) mismatches and ABO blood groups (see “Medical Background” Section). In our work, we increase the robustness of our privacy-preserving kidney exchange protocol by including the additional important biomedical factors from [13]. Furthermore, we recommend to use cycle sizes of two or three to reduce the impact of withdrawals [11]. The size is also beneficial for practical considerations with respect to medical staff and other resources needed for transplantations, as all operations of one exchange cycle should ideally be executed simultaneously. This recommendation reflects current best practices [14].

#### Privacy-preserving linear programming

Most currently used KEP solutions are based on Integer Linear Programming (ILP) formulations of the optimization problem. However, due to its superpolynomial complexity this is a largely unsolved space in the domain of privacy-preserving protocols. While multiple works considered secure linear programming using MPC (e.g., [15–17]), to our knowledge no results considering *integer* linear programming where some or all variables are not elements of a continuous field but must be integers. This research gap exsists for a good reason: Most exact ILP solving algorithms are based on “Branch and Bound” methods [18–20]. These methods find hyperplanes in the parameter space enclosing possible solutions, thus, pruning large sections of the parameter space. Unfortunately, a direct translation into the privacy-preserving realm would be vulnearble to timing attacks, hence insecure. Circuit-based MPC methods must exhibit deterministic runtimes, regardles of the specific inputs. Unfortunately, this disqualifies the privacy-preserving ILP approach for this work, as the presented algorithms inherently contain integer values in boundary conditions (e.g., encoding the graph structure).

### Privacy-preserving KEP protocols

Just two works, both by Breuer et al. [9, 10], investigate how to solve the kidney exchange problem in a decentralized privacy-preserving manner. Both consider the semi-honest security model.

### Privacy-preserving KEP protocol with HE

The first protocol [9] uses homomorphic encryption (concretely, a threshold variant of the Paillier cryptosystem [21]). It instantiates a computing party for each pair of a non-compatible donor and recipient at the providing hospital, thus, effectively creating a multi-party computation (MPC) protocol.

The protocol first pre-computes a set of all possible exchange constellations independent of any input data. Cycles of all lengths up to 3 are computed (but an arbitrary value could be chosen). Next, the pairs jointly compute an adjacency matrix with the pair-wise compatibility based on HLA crossmatching and ABO blood groups. Combining the results with the exchange constellations, the graph with the maximal size is delivered as the output. The protocol’s runtime scales exponentially with the number of pairs: starting with a runtime of 14 seconds for two pairs it increases to 13 h for nine pairs. Unfortunately, such runtimes are prohibitive for practical deployment.

### Privacy-preserving KEP protocol with Shamir’s secret sharing

In a concurrent work to ours, Breuer et al. [10] introduced a privacy-preserving KEP protocol for crossover kidney exchanges with polynomial computation complexity. “Crossover” hereby means that the kidney exchange is done among two pairs, i.e., the exchange cycle size is limited to two in contrast to [9]. This limitation, however, enables a significant efficiency improvement for matchings with more than 13 pairs. For example, with 15 pairs it reduces the runtime of the old protocol [9] from 8.5 h to 30 min. Additionally, the new protocol enables a dynamic setting, where donor-recipient pairs can be added/removed from the exchange graph at any point in time. On the technical side, the authors replace HE and fully rely on a MPC-technique called Shamir’s Secret Sharing (SSS) [22] implemented with the MP-SPDZ framework [23]. Beyond the dynamic setting and the change to MPC, the new protocol employs the graph matching algorithm by Pape and Conradt [24] for better efficiency in the matching between compatible donors and recipients.

Our privacy-preserving KEP protocol SPIKE offers significantly improved runtimes for real-world deployment. Our runtimes outperform the measured runtimes of previous works [9, 10], e.g., by a factor of hundreds/thousands for 9 recipient-donor pairs with a cycle length of 2. This is due to an efficient symbiosis of three MPC techniques and protocol optimizations that we will detail in the next section. Furthermore, we improve the robustness of SPIKE by including four additional biological factors notably impacting the transplantation success rate [13]. Thus, our protocol focuses on high medical quality rather than pure size, while also significantly improving efficiency.

## Background

In this work, we present a privacy-preserving solution to the *kidney exchange problem* (KEP). We interpret the KEP as an optimization problem, specifically finding cycles with a maximal coverage of nodes on a compatibility graph and a maximal aggregated edge weight. The graph is constructed according to medical compatibility factors. This section gives the required background information to understand the underlying aspects of biomedicine, graph theory, as well as the used privacy-preservation techniques of secure multi-party computation (MPC).

### Medical background

In the following, we introduce the medical background, i.e., biological factors used in our protocol that cause general immunological incompatibility or influence success likelihood for a kidney transplantation.

#### General immunological compatibility

While many medical factors are involved in the definite assessment of donor-recipient compatibility, some can be algorithmically determined. For example, one key factor in avoiding allograft rejection—immunological compatibility—can be evaluated following evidence-based guidelines. Our kidney exchange protocol uses a specific form of immunological compatibility, the HLA crossmatch, as a transplant prohibiting factor.

#### Human leukocyte antigens crossmatch

The human immune system is responsible for the protection of the organism against potentially harmful invaders (called *pathogens*). Antigens are molecular structures often found on the surface of pathogens, but also naturally occurring in the body. *Antibodies* can attach to those structures, preventing the pathogens from docking, thus inhibiting their harmful effect. One important group of endogenous antigens, which occur in varying numbers in every human, forming the immunological “fingerprint” the immune system recognizes as normal, are the *human leukocyte antigens*. Out of the three classes of HLA [25], only classes I and II are of interest in this work.

With a *HLA crossmatch* general compatibility between recipient and donor can be determined: The human leukocyte *antigens* of a donor are matched against existing human leukocyte *antibodies* of a possible recipient [26]. HLA crossmatch positive kidney transplants carry a significantly higher risk of antibody-mediated rejection or allograft rejection due to already existing antibodies [27, 28]. Modern immunosupressants might make such a procedure possible [29], but those cases require specialized, in-depth medical assessment and are out of scope of a general, algorithmic evaluation.

Following Eurotransplant’s guidelines [26], we consider HLA groups, which are also most frequently screened in preparation for kidney replacement therapy [30]: the HLA encoded at HLA-A, -B, and -DR loci. Additionally, we consider the HLA-DQ coded antigens, which are related to some cases of antibody-mediated rejection [31]. The full list of HLA considered in SPIKE can be seen in Table 1.

**Table 1:** HLA split antigens assessed for biomedical donor – recipient compatibility testing in SPIKE

Class I			Class II	
HLA-A	HLA-B		HLA-DR	HLA-DQ
A23	B38	B60	DR11	DQ5
A24	B39	B61	DR12	DQ6
A25	B44	B62	DR13	DQ7
A26	B45	B63	DR14	DQ8
A29	B49	B64	DR15	DQ9
A31	B50	B65	DR16	
A32	B51	B71	DR17	
A33	B52	B72	DR18	
A34	B54	B75		
A66	B55	B76		
A68	B56	B77		
A69	B57			
A74	B58			

#### Match quality estimation

Additionally to the previously introduced procedure that prevents immunological incompatibility, we strive to find the medically best/most robust solution to the kidney exchange problem – that includes maximal survival probability. For that, we calculate a match quality index, based on the following additional medical factors:

(i) *HLA match*
  Additionally to the HLA crossmatch, HLAs influence the probability of a successful transplantation. Concretely, it increases if the donor has a subset or the same HLA as the recipient. The number of “mismatches” is associated with increased allograft rejection rates, as the probability that a recipient develops antibodies to those mismatched antigens increases [32]. HLA mismatches do not constitute exclusion criteria, as immunosupressants can reduce the rejection probability. The use of immunosupressants, however, is itself linked to higher rejection rates [32–34]. Special importance comes to the HLA-DQ group, as mismatches of it are strongly linked to antibody-mediated rejections [31].
  Each person can inherit up to two types of HLA per group. Hence, at most two mismatches can occur per group [35]. The impact of HLA mismatches can be categorized in four bins: having no mismatch, a very rare case and mostly occurring in twin donor-recipient pairs, having 1–2 mismatches, having 3–4 mismatches, and, worst of all, having more than 5 mismatches [32]. The last group shows a more than 6% cumulative risk for death with a functioning graft during the first year. We weight HLA mismatches according to those four categories.
(ii) *ABO blood type*
  The ABO blood type system is based on the presence or absence of two types of antigens on the surface of the red blood cells [36]. The absence of both type A and type B antigens mark blood type O, the presence of both mark blood type AB, and the presence of only one mark blood type A and B, respectively. Receiving blood with an incompatible blood type leads to blood clumping due to an immune reaction and a possibly failed procedure. Compatible pairings are given in Table 2.
  By pre-processing the donor organ, grafts from ABO incompatible donors are possible [37], although linked to severe adversary reactions during the first year post transplantation. These reactions include a higher risk of allograft loss, severe viral infections, antibody-mediated rejections, and postoperative bleeding. After this first year, however, the long-term survival rate is comparable to ABO compatible transplants [37].
(iii) *Age*
  According to Waiser et al. [38], also age disparity influences allograft survival post transplant. The authors examined the role of age of the donor and recipient using two categories: *junior* participants aged below 55 years and *seniors* participants older than 55 years. The results show that intra-categorical transplants show the best outcomes, followed by pairings of junior donors and senior recipients. The worst outcomes were observed for pairings with senior donors and junior recipients.
(iv) *Sex*
  As shown by Zhou et al. [39], the combination of donor-recipient sexes impact the transplant success probability. The worst allograft survival rates were observed in male recipients for female donor organs, while same-sex pairs performed slightly better than female recipients for male donor organs.
(v) *Weight*
  Recipients, who received a kidney from a donor, who weighs less, have higher chances of allograft loss than other recipients [40]. El-Agroudy et al. [41] reason that the allograft loss for recipients with kidneys from lighter donors might be caused by the kidney being unable to support the body functions of a heavier recipient.

**Table 2:** ABO compatibility [36]

Blood group	Can receive from	Can donate to
O	O	O, A, B, AB
A	O, A	A, AB
B	O, B	B, AB
AB	O, A, B, AB	AB

### Graph theory

We represent the structure of the kidney exchange problem (KEP), as a (bipartite) graph problem. A graph $\mathcal {G}$ consists of a set of vertices $\mathcal {V}$ and an edge set $\mathcal {E}$ connecting the vertices. Technically, we deal with a *bipartite* graph, i.e., consisting of two different sets of vertices (donors and recipients), but as those register pairwise for the kidney exchange, we can “collapse” each donor-recipient pair into one vertex in $\mathcal {V}$. If two vertices $v, u \in \mathcal {V}$ are connected by an edge, then $(v, u) \in \mathcal {E}$. We consider a directed graph with directed edges from *v* to *u*. Furthermore, we use *weighted* edges by associating a scalar weight to each edge, according to its “importance” in the network. The weights represent the degree of medical compatibility. We only allow positive edge weights.

Our goal is to find all cycles within the graph. A cycle *c* is a list of vertices $\{v_1, v_{2}, ..., v_m\}$, where an edge exists from vertex $v_i$ to $v_{i+1}$ for $i \in \{1, ..., m-1\}$ and, to close the “loop”, from vertex $v_m$ back to vertex $v_1$. In a vertex disjoint cycle, each vertex appears at most once within the cycle. We define the length of a cycle as the number of edges that are used to form that cycle.

One representation of a (weighted) graph structure is the *adjacency matrix*, a square matrix *A* with one row/column for each vertex. If an edge exists between vertices *i* and *j*, then, the entry $a_{ij}=w$, with $w>0$ being the edge weight and $a_{ij}=0$ otherwise. This work uses the fact that by raising the adjacency matrix to the $\ell$th power, one can quickly compute the number of paths between two vertices with a given length $\ell$. That means, that vertices *i* and *j* are connected by $(A^\ell )_{ij}$ paths of length $\ell$. The diagonal elements give the number of cycles of length $\ell$ by finding paths starting and ending on the same vertex.

### Secure computation

Secure computation techniques enable multiple parties to securely evaluate an arbitrary function on their private inputs. Ideally nothing is leaked beyond what can be inferred from the output. A secure computation protocol must be able to realize this functionality without relying on a trusted party. To verify its security, it is typically compared to the so-called *ideal functionality*, which is a trusted third party that runs the computation on behalf of the data owners.

Privacy research has mainly worked on two paradigms for secure computation: Homomorphic Encryption (HE) and Secure Multi-Party Computation (MPC). HE schemes are special public-key encryption schemes that allow to realize (some limited) mathematical operations under encryption. However, they tend to be computing intensive making them (yet) often unsuitable for real-world applications. In contrast, MPC techniques are typically more efficient with respect to computation, as they are mainly based on efficient symmetric encryption and secret sharing. Additionally, MPC protocols can compute arbitrary functions. They are typically split into a setup and an online phase, where the setup phase is independent of the input data and, thus, can be pre-computed. This separation enables to significantly speed up the time-critical online phase as pre-computation can be done in idle times when input data is not yet available. However, MPC involves two or more parties, who jointly evaluate the desired function in a secure manner, hence, it requires communication among the parties. Both paradigms have already been used in the context of privacy-preserving genome-wide association studies [42–44], as well as other applications in the health care area [45–48].

To have provably secure privacy guarantees while achieving practical efficiency, SPIKE efficiently combines multiple MPC techniques, which we introduce in the following.

#### Secure multi-party computation (MPC)

Introduced by Andrew Yao’s seminarial work “How to Generate and Exchange Secrets” [49] in 1986, secure Multi-Party Computation (MPC) was considered a theoretical field first. MPC are cryptographic protocols that can securely compute an arbitrary function among two or more parties on their private inputs. Enabled by the rapid development of computer hardware and the development of the first MPC compiler “Fairplay” [50], first practical uses were demonstrated around the year 2004. Since then, MPC is a flourishing research field and due to novel protocols and optimizations, such as “Free $\texttt {XOR}$” [51] or “Halfgates” [52], practical applications in many fields were shown [45, 53, 54].

In this work, we rely on three well established secure *two-party* computation techniques, i.e., the secure computation protocols are run among exactly two parties: Arithmetic Secret Sharing ($\mathcal {A}$), Boolean Sharing ($\mathcal {B}$), both based on [55], and Yao’s Garbled Circuits (Y), originally introduced in [49]. Each technique differs in how it creates (*shares*) and reconstructs secrets, but also how (efficiently) certain types of operations can be realized.

#### Secure outsourcing

Although we use two-party MPC to perform the computation, *any* number of parties can provide input data. This method of *secure outsourcing* [56] works by all *data owners* secret sharing their data and sending one share to each of the two non-colluding *computation servers*. Secret sharing, thereby, means that the sensitive data is split into two random looking shares and each of the two computation servers receives only one of those. Specifically, a single computing server cannot infer any information about the secret input data from its share. Instead, the sensitive information can only be obtained when the secret shares of both servers are combined. The two computing servers, then, perform the actual secure computation on behalf of the data owners on the random looking secret shares, while not being able to learn anything about the private input data. To summarize, in the outsourcing scenario an arbitrary number of data owners can participate without leaking/uploading *any* sensitive information to an external party.

This scenario has three main benefits:

- The communication of *N*-party MPC scales at least linearly, often quadratic in the number of participating parties [57]. By outsourcing the *N*-party computation to $M \ll N$ parties, here $M=2$, the complexity is improved substantially.
- As the input owners do not participate in the computation itself, the outsourced protocol provides security against malicious data owners [56]. At most they can corrupt the correctness of the calculation, but not the privacy.
- The location of the computation servers can be chosen pragmatically, e.g., two locations with high bandwidth and low latency network connection. Of course, the computation servers are assumed to not collude.
- Compared to *N*-party MPC setups, two-party MPC requires to trust exactly one computation server. A data owner can also run one himself. Using $N>2$ non-colluding parties can be more efficient [58, 59], but ensuring the non-collusion among all *N* parties is more challenging/might not be realistic. Full threshold *N*-party MPC schemes [60], i.e., where all but one party can be compromised, significantly reduces efficiency/increase communication.^4^ To summarize, outsourcing to two non-colluding servers offers a good trade-off between efficiency and security.

#### Security model

In our work, we consider the *semi-honest* security model, where the two computation servers are assumed to be honestly following the protocol, while trying to learn as much information as possible. By “honestly following the protocol” we, thereby, mean that they adhere to the specifications of the protocol, e.g., they do not manipulate local calculations or provide inconsistent data. Additionally, the two computation servers are assumed to not collude. This security model provides protection against curious personnel or accidental data leakage and the omission of a trusted third party further reduces the impact of a potential data breach. Although weaker than the malicious security model, where the parties might arbitrarily deviate from the protocol, the semi-honest security model is sufficient for our use case, as hospitals are generally trusted, but legally not allowed to simply share the data among each other. Furthermore, the semi-honest security model enables significantly more efficient computation than the malicious model and, hence, provides a good efficiency-privacy trade-off. While the European Data Protection Board recommends security against malicious adversaries when performing joint calculations with parties under jurisdiction of insecure countries [62], the semi-honest security model is the predominant model in data protection concepts for federated medical research^5^. Hence, it is a valuable security model in our application scenario. Previous works on privacy-preserving KEP protocols [9, 10] are also in the semi-honest security model.

#### Notation

In the following, $\langle x \rangle _{i}^s$ denotes a secret share of *x* shared using MPC technique $s\in \{A,B,Y\}$ and held by party $P_i$, where $i\in \{0,1\}$.

#### Yao’s garbled circuits

($$\mathcal {Y}$$)

Yao’s Garbled Circuits enable two parties, called the *garbler* and the *evaluator*, to securely evaluate a function *f* represented as *Boolean circuit*, i.e., a directed acyclic graph, where the nodes are logic gates and the edges (called *wires*) are the Boolean in- and outputs. For functional completeness $\texttt {AND}$ and $\texttt {XOR}$ gates are sufficient. The garbler generates random keys for each possible state of each wire $k^w_0,k^w_1\in \{0,1\}^\kappa$, where $\kappa$ is the symmetric security parameter (set to $\kappa =128$ in our implementation) and *w* is the respective wire. For all input combinations of each gate in the circuit, it uses the input keys to encrypt the corresponding output key (cf. Table 3). The order of the four ciphertexts is then permuted randomly and the *garbled circuit* is sent to the evaluator together with the keys associated to the garbler’s input. As those keys look random, the evaluator cannot extract any information about the input of the garbler. Next, the evaluator engages in an oblivious transfer [65, 66] to receive the keys for his input without revealing it to the garbler. Equipped with all keys, it evaluates the garbled circuit to receive the circuit’s output keys, which the parties jointly decode. Thanks to several optimizations, e.g., [51, 52, 67], $\mathcal {Y}$ requires no communication for the evaluation of an $\texttt {XOR}$ gate and only $1.5\kappa$ bits of communication for $\texttt {AND}$ gates. $\mathcal {Y}$ needs a constant number of communication rounds independent of the circuit depth.

**Table 3:** Garbled AND gate

Input $w_0$	Input $w_1$	Output $w_2$	Garbled value
$k_0^{w_0}$	$k_0^{w_1}$	$k_0^{w_2}$	$\mathrm {Enc}_{k_0^{w_0},k_0^{w_1}}(k_0^{w_2})$
$k_0^{w_0}$	$k_1^{w_1}$	$k_0^{w_2}$	$\mathrm {Enc}_{k_0^{w_0},k_1^{w_1}}(k_0^{w_2})$
$k_1^{w_0}$	$k_0^{w_1}$	$k_0^{w_2}$	$\mathrm {Enc}_{k_1^{w_0},k_0^{w_1}}(k_0^{w_2})$
$k_1^{w_0}$	$k_1^{w_1}$	$k_1^{w_2}$	$\mathrm {Enc}_{k_1^{w_0},k_1^{w_1}}(k_1^{w_2})$

#### Boolean and arithmetic secret sharing

($$\mathcal {B}$$/$$\mathcal {A}$$)

In Additive Arithmetic Secret Sharing ($\mathcal {A}$) operations on $\ell$-bit inputs are done in an algebraic ring ${\mathbb {Z}}_{2^{\ell }}$, where $\ell$ is the bit length. Although the technique can also be used among an arbitrary number of parties [68], we focus here on the two party setting as introduced by Goldreich et al. [55].

To share a secret value *x*, party $P_i$, $i\in \{0,1\}$, generates a random value $r\in _R {\mathbb {Z}}_{p}$ and sets its arithmetic share to $\langle x\rangle ^A_{i}=r$. Then, $P_i$ also determines party $P_{1-i}$’s share $\langle x\rangle ^A_{1-i}=x-r \mod 2^{\ell }$ and sends it to $P_{1-i}$. To reconstruct the secret, one needs to know *both* shares and compute $x=\langle x\rangle ^A_0+\langle x\rangle ^A_1 \mod 2^{\ell }$. Boolean Secret Sharing ($\mathcal {B}$) describes the special case, where $\ell =1$, viz. ${\mathbb {Z}}_2=\left\{ 0,1\right\}$.

Note that a share $\langle x\rangle ^A_i$ (resp. $\langle x\rangle ^B_i$) is random and does not leak anything about the secret *x*. Secure addition (respectively, $\texttt {XOR}$ing in $\mathcal {B}$) can be executed locally, that is without communication between the parties. Secure multiplication (respectively, $\texttt {AND}$ in $\mathcal {B}$) is done in an interactive protocol among the two parties using so-called multiplication triples [61, 69, 70]. Using only addition and multiplication (similarly, $\texttt {AND}$ and $\texttt {XOR}$) arbitrary functions can be calculated.

#### ABY framework

All three MPC techniques are implemented in the state-of-the-art secure two-party computation framework ABY [61], which we use in our experiments^6^. Additionally, ABY also contains efficient conversions between them and supports Single Instruction Multiple Data (SIMD) operations to parallelize identical operations on different data, while reducing memory usage and runtime. Arithmetic Secret Sharing in ABY is performed on the ring ${\mathbb {Z}}_{2^\ell }$, that is with $2^\ell$ elements, where $\ell$ is the bitlength of the data type (most often $\ell = {32}{\hbox {bit}}$). A recent work by Patra et al. [53] improves [61] by making the online communication of scalar multiplication independent of the vector dimensions and reducing online communication for $\texttt {AND}$ gates with two inputs in $\mathcal {B}$ by a factor of 2. Unfortunately, these protocols have been implemented only very recently in MOTION2NX [72], which is why we use [61] in our implementation.

## Methods

In this section, we first define the privacy-preserving Kidney Exchange problem (KEP) and its requirements. Then, we present our solution, which we name SPIKE, consisting of tailored modular secure MPC protocols and include a complexity analysis.

### Problem statement

**Fig. 2:**
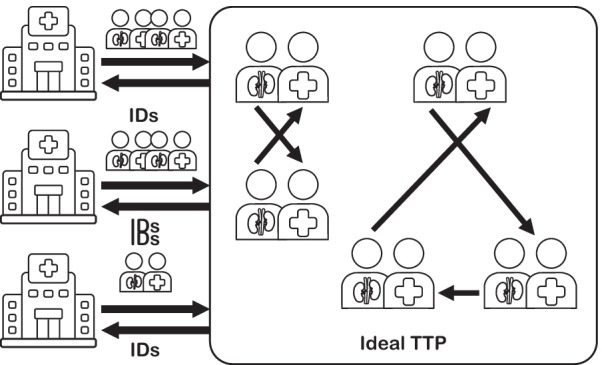
Ideal functionality for a secure privacy-preserving protocol solving the kidney exchange problem (KEP)

Figure 2 shows the ideal functionality for solving the privacy-preserving KEP in a provably secure way. Assuming the (not realistic) availability of a trusted third party (TTP), hospitals send the data of recipients and donors to the TTP, which calculates cycles of pairs of recipients and donors with the highest probability to be compatible. Then, the TTP returns for each recipient the information about his/her donor to the respective hospital. Note that a final evaluation must still be done by medical experts. A privacy-preserving KEP protocol is meant for automatizing and, thus, accelerating, the process of the creation of the kidney exchange cycles.

*Requirements* We define the following requirements for a secure privacy-preserving KEP protocol:

- *Privacy* The privacy-preserving KEP protocol must realize the same functionality as described in the ideal functionality, while removing the problematic assumption of a TTP, i.e., it must leak nothing beyond what can be inferred from the output.
- *Efficiency* The privacy-preserving KEP protocol must be efficient in terms of communication and computation, such that it can be run in reasonable time on standard server hardware.
- *Decentralization* The privacy-preserving KEP protocol must be decentralized, i.e., the highly sensitive medical information of donors and patients must remain locally at the respective medial institution (inherently being compliant with the data minimisation principle).
- *Adaptability for Medical Experts* The priva-cy-preserving KEP protocol must be flexibly adaptable for medical experts with respect to the selection of biological factors for the algorithmic evaluation of compatibility. They must be able to adjust the weighting between the included factors and cycle lengths according to state-of-the-art medical advancements. The protocol must be easily extendable to new factors and additional HLA groups.

### SPIKE: a MPC-based privacy-preserving KEP protocol

In this section, we provide the building blocks for our Secure and Private Investigation of the Kidney Exchange problem: SPIKE. It fulfills above requirements (see also the overview of the phases in Fig. 1).

First, we explain the matching phase, which analyzes the compatibility between donors and recipients using six biological factors presented in the "Background" section. Then, we continue with the determination of the number of potential exchange cycles given a cycle length. The third phase computes the probability of a successful transplantation based on the matching results for all potential exchange cycles. In the final phase, we output a robust set of *disjoint* exchange cycles, i.e., with a high probability for compatibility. The final result contains a combination of disjoint exchange cycles that maximizes the likelihood of as many transplantation as possible being successful. The weight of a cycle *c* is denoted by $w_c$ where a higher value indicates a higher likelihood of a transplantation being successful. Thus, the weight of a set of disjoint cycles $\mathcal {C}$, i.e., the likelihood for as many transplantation being successful in the set, can be described as the sum of all cycles $w_{c_i}$ for $i \in \{1, \ldots , |\mathcal {C}|\}$ in the set. The weight of a cycle is determined by the sum over all edge weights $w_e$ in the cycle. Finally, the weight of an edge is determined by the sum over the results of all matching criterion which are multiplied by a weight which can be assigned by medical experts to highlight certain biomedical factors. Note that we write this computation as a dot product between a vector $\vec {p}(k, l)$ and $\vec {w}$ where $\vec {p}(k, l)$ contains the results of the matching between pair *k* and *l* in vector form and $\vec {w}$ the respective weights of each criterion. Equation (1) describes the previous conditions. To achieve the described result, we greedily select disjoint cycles in decreasing priority according to the weight of each individual cycle.

1 $$\begin{aligned}&\max \sum _{i=1}^{|\mathcal {C}|} \overbrace{\left( \sum _{j=1}^{|cLen|} \underbrace{\left( \vec {p}(k, l) \cdot \vec {w} \right) _j}_{:=w_{e_j}} \right) _i}^{:=w_{c_i}}\qquad&\end{aligned}$$

Note that our solution is a local optimum which is computed with a greedy algorithm while the solutions by Breuer et al. [9, 10] are globally optimal. We argue that a locally optimal solution is sufficient in our application scenario for two reasons: First, we assume that the locally optimal results are in close proximity of the global optimum, as real world data sets will likely show sparse compatibility and the additional medical compatibility factors considered by SPIKE will increase the solution quality. Second, the additional expert evaluation following the algorithmic matching will most likely introduce a much higher variance in the chosen solution. The empirical evaluation of those two claims are interesting points for further research requiring real-world kidney exchange data sets. The protocol presented by Breuer et al. [10] enables usage in a dynamic setting, i.e., a setting in which donor-recipient pairs are put together in a pool where pairs come and go over time. They run their matching protocol on a subset of the pairs of the pool and, afterwards, evaluate the resulting compatibility graph. By design, SPIKE enables usage in a dynamic setting, too, since each part of the protocol can be executed independently of the others parts as long as they receive the output of the previous parts. Such a dynamic setting can also be adapted to an outsourcing scenario. Each input party has their own pool of donor-recipient pairs where they can select a random subset of pairs and send them to the computing parties.

#### Notation

We use Boolean operators to concisely present our MPC protocols: $\wedge$ is $\texttt {AND}$, $\vee$ is $\texttt {OR}$, $\lnot$ is $\texttt {Not}$, and $\oplus$ is $\texttt {XOR}$. 0/1 are False/True. |*x*| indicates the length of a vector *x*, i.e., the number of elements. Non trivial variable names in protocols are written in $\mathsf {sans~serif}$, function names (and calls) $\texttt {monospaced}$. Branching, implemented with $\texttt {MUX}$ (multiplexer) Gates, is displayed using ternary notation: condition **?** true statement **:** false statement.

#### Compatibility matching

The first phase of SPIKE is called *compatibility matching*. In this phase, we compare the pair-wise general compatibility and match quality of all donors and recipients with respect to human leukocyte antibodies and antigens, ABO blood group compatibility, age, sex, and weight. The output of this phase is a weighted compatibility graph, where the edge weights indicate the probability of compatibility for each pair.

**Table 4:**
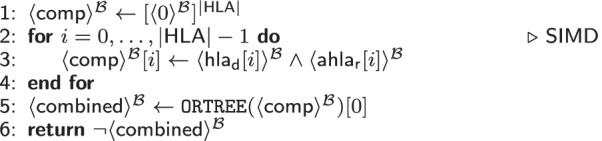
$\texttt {matchHLA}(\langle \mathsf {hla_d}\rangle ^{\mathcal {B}}$: vector, $\langle \mathsf {ahla_r}\rangle ^{\mathcal {B}}$: vector) $\rightarrow$ int

We present the main protocols for the compatibility assessment in the following. The subprotocols for assessing the individual matching criteria HLA mismatches, ABO blood type, age, sex, and weight are given as Additional file 1: Tables S1–S6 in the Appendix.

The HLA crossmatch subprotocol is shown in Table 4. It tests whether the human leukocyte antigens of the donor are unsuitable to the human leukocyte antibodies of the recipient rendering them incompatible.

The subprotocol takes a vector with the antigens of a donor $\mathsf {hla}_d$ and a vector with the antibodies of the recipient $\mathsf {ahla}_r$ as input. The number of observed HLA, denoted by $|\mathsf {HLA}|$, is publicly known. A vector $\mathsf {comp}$ stores whether the recipient possesses an antibody against any of the donor’s HLA (cf. Line 3). For enhanced efficiency, we parallelize this comparison as *Single Instruction, Multiple Data* (SIMD) operation, such that all HLA matches of one patient are computed in just one step. Afterwards, the overall compatibility (i.e., no antigen-antibody mismatch was found) is computed with $\texttt {OR}$ gates in a tree structure, to reduce the (multiplicative) dephts of the circuit from $|\mathsf {HLA}|$ to $\log _2(|\mathsf {HLA}|)$. To prepare for further processing, we invert $\mathsf {combined}$ and return it as result of the HLA crossmatching in Line 6.

**Table 5:**
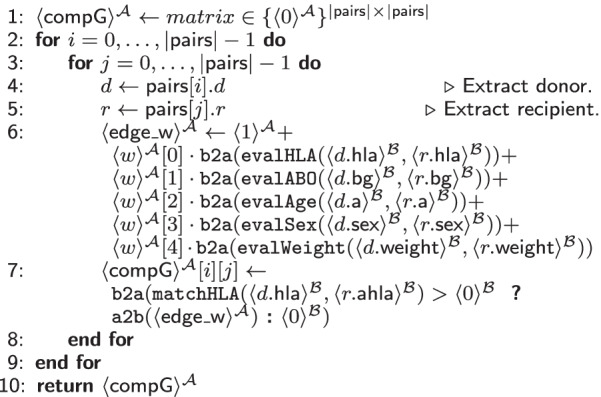
$\texttt {computeCompatibilityGraph}(\langle \mathsf {pairs}\rangle ^{\mathcal {B}}$: vector, $\langle \mathsf {w}\rangle ^{\mathcal {A}}$: vector) $\rightarrow$ weighted adjacency matrix

In Table 5, we present our MPC protocol that combines the results of the evaluated six medical criteria influencing the compatibility of a kidney donation into a weighted adjacency matrix indicating the donor-recipient compatibility, named $\mathsf {compG}$.

It takes a vector $\mathsf {pairs}$ containing all possible pairs of donors and recipients and a vector *w* with a weight for each criteria (i.e., how much it influences the overall probability for good compatibility compared to the other factors) as input. Lines 4 to 6 additively combine the computed weighted probability of each compatibility criterion and assign it to the respective edge representing the donor of the *i*-th pair and the patient of the *j*-th pair, where $i\ne j$ and $i,j\in \{0,\ldots ,|\mathsf {pairs}|-1\}$. In Line 7, we additionally check whether the *i*-th donor and the *j*-th patient exhibit general immunological compatibility using the HLA crossmatch subprotocol (cf. Table 4). If this is the case, we store the result of the edge weight at the respective index, otherwise, we store the secret shared constant 0.

*MPC Cost.* The two sections in Table 4 evaluate $|\mathsf {HLA}|$ $\texttt {AND}$ gates (as SIMD) and $\log _2(|\mathsf {HLA}|)$ $\texttt {OR}$^7^ gates, respectively. Finally, we invert $\mathsf {combined}$ once. This results in a circuit depth of $\log _2(|\mathsf {HLA}|)+1$ and a total number of $\texttt {AND}$ gates of $2\times |\mathsf {HLA}|$. Boolean sharing ($\mathcal {B}$) is used in this protocol, as Boolean operations are performed and the circuit depths is low, thanks to the SIMD vectorization [61].

To fully assess the matching quality (Table 5), all criteria have to be evaluated for each recipient, i.e., Table 4 and Additional file 1: Tables S1, S2, S4, and S6 are run $|\mathsf {pairs}|^2$ times. Then, in Table 5, we additionally evaluate five multiplications, five additions, one comparison, one $\texttt {AND}$ gate, and one $\texttt {MUX}$ gate. Due to the arithmetic operations in this protocol, the results of the compatibility evaluation protocols must be converted between $\mathcal {B}$  and $\mathcal {A}$.

#### Cycle computation

The second phase of SPIKE computes the number of possible kidney exchange cycles given a concrete input cycle length^8^ from the compatible donors and recipients that were output by the compatibility matching. Our MPC protocol for this part is shown in Table 6.

**Table 6:**
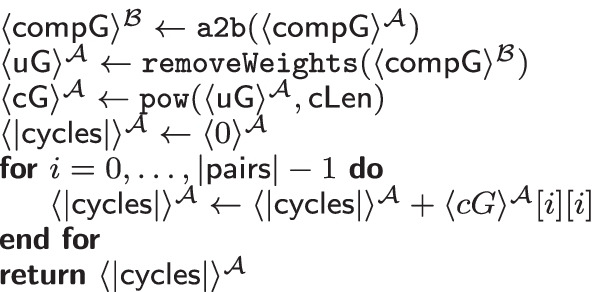
$\texttt {determineNumberCycles}(\langle \mathsf {compG}\rangle ^{\mathcal {A}}$: matrix) $\rightarrow$ number of cycles

Table 6 takes the secret shared weighted compatibility graph $\mathsf {compG}$ as input. The desired length of cycles $\mathsf {cLen}$ is public. We first compute the unweighted adjacency matrix in Line 2 (cf. Additional file 1: Table S7, in the Appendix). For the unweighted matrix, we compute the $\mathsf {cLen}$-th power using a naïve implementation^9^. The entries in this resulting matrix indicate how many paths of length $\mathsf {cLen}$ start at vertex *i* and end at vertex *j*. For cycles, the entries are on the diagonal, as start- and end-vertex are identical. Following this thought, the sum of the entries of the diagonal is the total number of cycles with the given cycle length $\mathsf {cLen}$. Note that this number contains duplicates, namely, “congruent” cycles that are the same, but were found via a different start/end vertex.^10^ We remove the duplicates later in Additional file 1: Table S9 (described in the Appendix).

*MPC Cost.* Table 6 contains mostly arithmetic operations ($|\mathsf {pairs}|^3$ multiplications and $(|\mathsf {pairs}|^3-|\mathsf {pairs}|^2$ additions), however, the computation of the unweighted adjacency matrix is most efficiently performed in $\mathcal {B}$ $|\mathsf {pairs}|^2$ comparisons and $\texttt {MUX}$ gates). For that reason we convert $\mathsf {compG}$ from $\mathcal {A}$ to $\mathcal {B}$ (cf. Line 1) and back (in Additional file 1: S7).

**Table 7:**
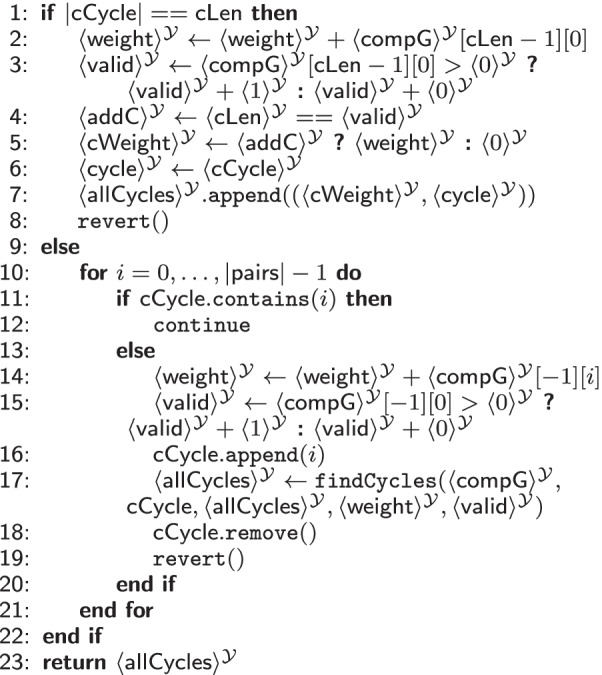
$\texttt {findCycles}(\langle \mathsf {compG}\rangle ^{\mathcal {Y}}$: matrix, $\mathsf {cCycle}$: vector, $\langle \mathsf {allCycles}\rangle ^{\mathcal {Y}}$: vector, $\langle \mathsf {weight}\rangle ^{\mathcal {Y}}$: int, $\langle \mathsf {valid}\rangle ^{\mathcal {Y}}$: int) $\rightarrow$ vector of tuples

#### Cycle evaluation

The third phase of SPIKE then identifies the most likely successful unique exchange cycles consisting of compatible pairs of donors and recipients and sorts them in descending order with respect to their weight.

Our first subprotocol for this phase, shown in Table 7, finds all exchange cycles of the desired length (including duplicates) and computes the weight of each cycle. This weight is the sum of all included weighted edges. As mentioned before, the weight associated with an exchange cycle indicates the probability of the transplantation being successfully carried out, i.e., its robustness.

The subprotocol takes the secret shared compatibility graph $\mathsf {compG}$ output by Table 5, the currently analyzed exchange cycle $\mathsf {cCycle}$, its secret shared weight $\mathsf {weight}$, a secret shared counter $\mathsf {valid}$, which tracks the number of edges in $\mathsf {cCycle}$, and a vector of secret shared tuples $\mathsf {allCycles}$, which will be consecutively filled with all possible exchange cycles and the corresponding sum of weights. In a recursive execution of Subprotocol 7, this vector is filled, as explained in detail in the following. The desired output cycle length $\mathsf {cLen}$ and the number of recipient-donor pairs $|\mathsf {pairs}|$ are public. Contrary to the protocols in [9, 10], the output number of cycles $|\mathsf {cycles}|$ found in Table 6 is revealed for efficiency reasons. We consider this leakage as acceptable since it leaks only a very high-level aggregate property, generally not allowing the inference of the compatibility graph’s topology^11^. In the legal sense, the revealed number is considered non-sensitive as well, as it is an aggregated, anonymized datum.

Table 7 first checks if the currently analyzed exchange cycle $\mathsf {cCycle}$ already has the desired length $\mathsf {cLen}$. If this is the case, the weight of the last edge is added to the respective sum of this cycle’s weights in Line 2. Next, each valid $\mathsf {cCycle}$ is added to $\mathsf {allCycles}$ with its respective sum of weights. A $\mathsf {cCycle}$ is valid, if it is closed (cf. Lines 3–4). An invalid cycle is associated with weight zero (cf. Line 5). Note that a weight of zero does not contribute to the solution, hence a cycle with weight zero is never considered for a solution. In Line 8, the operations done in Lines 2–3 are reverted to restore the state of $\mathsf {cCycle}$ before the last edge was added, i.e., the weight of the last edge is subtracted from $\mathsf {weight}$ and $\mathsf {valid}$ is decreased by 0 (no edge) or 1 (edge).

Cycles that do not have the desired length yet are handled in Lines 10–21. For these exchange cycles, the subprotocol checks whether they are already part of $\mathsf {cCycle}$, as each vertex may only appear at most once (cf. Line 11). If it is not included, the weight of the edge from the previous to the new vertex is added by increasing $\mathsf {cCycle}$’s weight and counter $\langle valid \rangle ^{\mathcal {Y}}$, and the new vertex is added to $\mathsf {cCycle}$ (cf. Lines 14–16). Afterwards, Table 7 is recursively called again with the newly added vertex. Once the function returns, we revert the operations done before to be able to analyze the next cycle (cf. Lines 18–19).

The second subprotocol of the cycle evaluation (cf. Additional file 1: Table S9 in the Appendix) removes duplicates from the exchange cycles set. It extracts $\#{\mathsf {unique}}=\lfloor \frac{\#{\mathsf {cycles}}}{\mathsf {cLen}} \rfloor$ cycles and returns the *k* cycles with the highest probability for a successful transplantation.

**Table 8:**
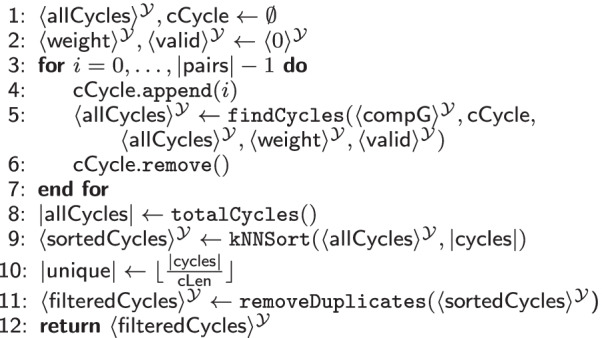
$\texttt {evaluateCycles}(\langle \mathsf {compG}\rangle ^{\mathcal {Y}}$: matrix) $\rightarrow$ vector of tuples

Table 8 combines the previously discussed subprotocols. It first calculates the sum of weights for each cycle with Table 7 (findCycles) and sorts the result using Additional file 1: Table S8 (kNNSort), such that only the *k* cycles with the largest weight are output. Those are all valid cycles, possibly including duplicates. Afterwards, the protocol removes all duplicates within the *k* cycles.

*MPC Cost.* The complexity of Subprotocol 7 depends on the number of pairs $|\mathsf {pairs}|$, $\mathsf {cLen}$, and the number of possible cycles $|\mathsf {allCycles}|$. It is most efficient in $\mathcal {Y}$ , as the $\texttt {MUX}$ gates are not independent, thus, creating a deep circuit of depth $\mathcal {O}(|\mathsf {allCycles}| \times |\mathsf {cycles}| \times \mathsf {cLen})$. For removing duplicates and extracting the most robust *k* exchange circuits, we evaluate $\#{\mathsf {cycles}}\times (\#{\mathsf {unique}}+\sum _{i = 0}^{\#{\mathsf {cycles}}}\left( \mathsf {cLen} \times (\mathsf {cLen}-1)\right)$) comparisons, $\#{\mathsf {cycles}}$ $\times$ $\sum _{i = 0}^{\#{\mathsf {cycles}}}((\mathsf {cLen} \times (\mathsf {cLen}-1)))$ $\texttt {AND}$ gates, $\#{\mathsf {cycles}}$ $\times$ $\sum _{i = 0}^{\#{\mathsf {cycles}}}\left( \mathsf {cLen}-1\right)$ $\texttt {OR}$ gates, $\#{\mathsf {cycles}}$ $\times \#{\mathsf {unique}} \times (1 + \mathsf {cLen}) + \#\mathsf {cycles}$ $\texttt {MUX}$ gates. This step is most efficient with $\mathcal {Y}$ , as the circuit is very deep. Thus, the complete cycle evaluation routine is most efficient in $\mathcal {Y}$ , as each of our subroutines is most efficient in $\mathcal {Y}$.

#### Solution evaluation

The fourth phase of SPIKE determines the final output, a set of *disjoint* exchange cycles exhibiting the highest probability for a successful transplantation. As a pair of donor and recipient can only be involved in one exchange cycle, the output sets must be vertex disjoint. Thus, the resulting set contains a combination of disjoint exchange cycles that greedily maximizes the number of exchanges with respect to the likelihood of the transplantation being successful. Note that we find a locally optimal solution, which might differ from the globally optimal solution^12^. The locally optimal solution is computed using a greedy algorithm. This last part of SPIKE is shown in Table 9.

**Table 9:**
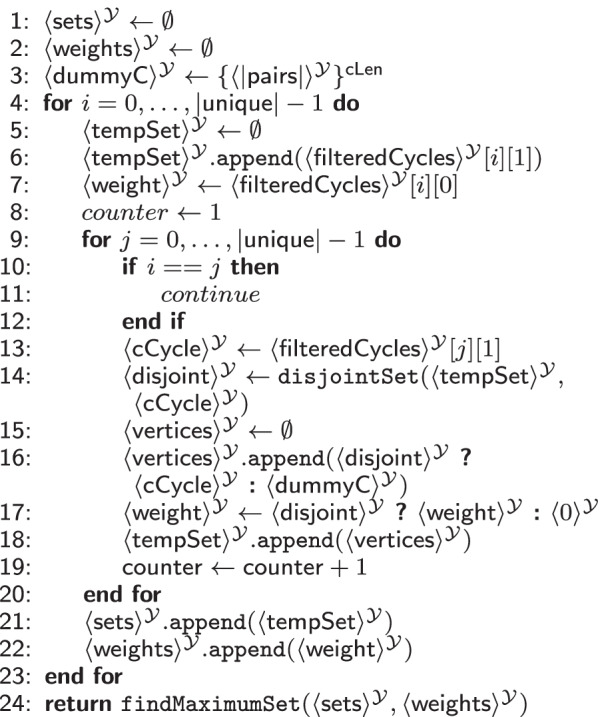
$\texttt {evalSolution}(\langle \mathsf {filteredCycles}\rangle ^{\mathcal {Y}}$: vector of tuples) $\rightarrow$ tuple(int, vector of vectors)

Table 9 takes a secret shared vector of tuples $\mathsf {filteredCycles}$ with all valid unique cycles and their respective weights, the number of valid cycles $|\mathsf {unique}|$, and the cycle length $\mathsf {cLen}$ as input. The number of pairs $|\mathsf {pairs}|$ is a public variable as before.

It checks each valid cycle $\mathsf {cCycle}$ whether it is disjoint from all other previously analyzed cycles in $\mathsf {tempSet}$. The MPC subprotocol for testing the disjointness is given in Additional file 1: S11 in the Appendix. If it is disjoint, $\mathsf {cCycle}$ is added to the set of potential solutions (Lines 16–22). Finally, the set with the highest weight is returned. Details of the corresponding MPC protocol can be found in Additional file 1: Table S12 in the Appendix.

*MPC Cost.* In total, we evaluate $|\mathsf {unique}|^2$ $\texttt {ADD}$ gates, $|\mathsf {unique}|^2 \times \mathsf {cLen}^2 + |\mathsf {unique}|$ comparisons, $4 \times |\mathsf {unique}|^2 + |\mathsf {unique}|$ $\texttt {MUX}$, and $|\mathsf {unique}|^2 \times \mathsf {cLen^2}$ $\texttt {OR}$ gates. The solution evaluation is most efficient in $\mathcal {Y}$ , as there are only few arithmetic operations and mostly comparisons.

### Complexity assessment

In Table 10, the asymptotic complexities for the four phases of SPIKE are given.

**Table 10:** Complexity assessment

Phase	Name	Protocol	Time complexity
Part 1	Compatibility matching	Table 4	$\mathcal {O}(|\mathsf {HLA}|)$
		Additional file 1: Table S1	$\mathcal {O}(|\mathsf {HLA}|)$
		Additional file 1: Table S2	$\mathcal {O}(1)$
		Additional file 1: Table S4	$\mathcal {O}(1)$
		Additional file 1: Table S5	$\mathcal {O}(1)$
		Additional file 1: Table S6	$\mathcal {O}(1)$
		Table 5	$\mathcal {O}(|\mathsf {pairs}|^2 \times |\mathsf {HLA}|)$
Part 2	Cycle computation	Additional file 1: Table S7	$\mathcal {O}(|\mathsf {pairs}|^2)$
		Table 6	$\mathcal {O}(\mathsf {cLen} \times |\mathsf {pairs}|^{3})$
Part 3	Cycle evaluation	Additional file 1: Table S10	$\mathcal {O}(1)$
		Table 7	$\mathcal {O}(|\mathsf {pairs}|^{\mathsf {cLen}})$
		Additional file 1: Table S8	$\mathcal {O}(|\mathsf {cyclesSet}| \times \mathsf {k} \times \mathsf {cLen})$
		Additional file 1: Table S9	$\mathcal {O}(|\mathsf {cycles}|^2)$
		Table 8	$\mathcal {O}(|\mathsf {pairs}|^{\mathsf {cLen}})$
Part 4	Solution evaluation	Additional file 1: Table S11	$\mathcal {O}(|\mathsf {cycles}|\times \mathsf {cLen})$
		Additional file 1: Table S12	$\mathcal {O}(\mathsf {cycles}|^{2})$
		Table 9	$\mathcal {O}(|\mathsf {cycles}|^{3} \times \mathsf {cLen^2})$

The most important parameters of the first part, the Compatibility Matching shown in the first section of the table, are the number of HLA (cf. Background) $|{\mathsf {HLA}}|$ and the number of pairs $|{\mathsf {pairs}}|$. In the default configuration, $|\mathsf {HLA}|$ is 50. For the second phase, the dominant parameter is the number of pairs $|\mathsf {pairs}|$. In the third section of Table 10, the asymptotic complexity for the Cycle Evaluation is given. The relevant parameters here are the number of pairs $|\mathsf {pairs}|$, the total number of cycles $|\mathsf {allCycles}| = |\mathsf {pairs}|^{\mathsf {cLen}}$, the number of existing cycles $|\mathsf {cycles}|$, the number of unique cycles $|\mathsf {unique}| = \lfloor \tfrac{|\mathsf {cycles}|}{\mathsf {cLen}} \rfloor$, the length of cycles $\mathsf {cLen}$, and the factor *k* (i.e, the number of cycles with highest probability for successful transplantation), and the number of elements in $\mathsf {cyclesSet}, |\mathsf {cyclesSet}|$ of Table 8. The most important parameters of the last phase, the Solution Evaluation, are the number of unique cycles $|\mathsf {cycles}|$, and the length of cycles $\mathsf {cLen}$.

Overall, the asymptotic complexity of SPIKE is:

$$\begin{aligned} \mathcal {O}(|\mathsf {pairs}|^2 \times |\mathsf {HLA}| + \mathsf {cLen} \times |\mathsf {pairs}|^{3} + |\mathsf {cycles}|^3 \times \mathsf {cLen}^2). \end{aligned}$$

The most most important parameters are the number of pairs $|\mathsf {pairs}|$, the number of considered HLA $|\mathsf {HLA}|$, the length of cycles $\mathsf {cLen}$, and the number of unique cycles $|\mathsf {cycles}|$.

## Results

All benchmarks were run on two servers equipped with Intel Core i9-7960X processors and 128 GB RAM. They are connected via 10Gb/s LAN with a median latency of 1.3ms. All benchmarks are averaged over 10 runs.

### Network setups

To provide meaningful performance benchmarks for a variety of real-world settings, we envision two network settings for the privacy-preserving KEP protocol that we describe in the following. In addition, for the comparison to the works of Breuer at al. [9, 10], we replicated their network setting with 1Gb/s bandwidth and 1ms of latency.

#### LAN

The high-bandwidth, low latency network scenario, here referred to as *LAN*, is the most relevant real-world scenario for our application. In Germany, most (larger) medical institutions utilize high-bandwidth Internet connections. In the case of most university hospitals the German Research Network (“Deutsches Forschungsnetz” DFN^13^) provides dedicated, high bandwidth communication networks. Our *LAN* benchmarks are performed using a 10Gb/s connection with an average latency of 1.3ms.

#### WAN

One benefit of a MPC-based privacy-preserving KEP solution could be reduced legal and regulatory data protection requirements, due to the high security level of the computation itself. This would allow smaller, local hospitals and medical practices to directly participate in the kidney exchange. Those institutions might be connected via residential Internet access. For that scenario, we benchmarked SPIKE in a reduced-bandwidth, high latency network. A bandwidth restriction to 100Mb/s with added latency of 100ms was implemented using the tc^14^ command to simulate the *WAN* network. The high latency was chosen to take packet loss due to unreliable connections into account.

### Performance benchmarks

**Fig. 3:**
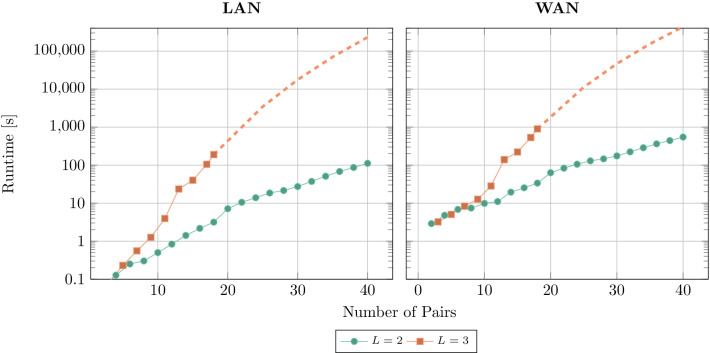
Overall runtime of SPIKE for cycle lengths $L=2$ and $L=3$ in both network scenarios. The dashed line shows the extrapolated power function for $L=3$

Figure 3 shows the total runtime of SPIKE for varying numbers of pairs, both network settings, and cycle lengths $L=2$ and $L=3$. The full results are in the Additional file 1: Tables S13–S20 in the Appendix.

During the evaluation of longer cycles ($L\ge 3$) RAM utilization proved itself to be a bottleneck for execution. For those scenarios, we benchmarked up to RAM exhaustion and extrapolated the runtimes according to the underlying power-law complexity. The extrapolation is shown with a dashed line. The sudden increase in runtime for $L=3$ between 12 and 13 pairs occurs due to swapping.

As a general result, the expected polynomial relationship between the number of pairs and the overall runtime can be observed, reflected in the power-law development in the semilog graphs. For $L=2$, we achieve a total runtime of under 4min for 40 pairs, thus, demonstrating real-world applicable performance. The WAN setting increases the overall runtime by less than an order of magnitude. Calculation times under 20min for 40 pairs in this setting render the participation feasible for physicians with residential Internet connections. To find a solution for larger cycle lengths, the exponent in the time complexity increases, increasing the runtimes significantly. But even then 25 pairs are computable in around 1 h. Extrapolated to data set sizes of 100 pairs, SPIKE is able to finish the calculation for cycle length $L=2$ in just over 2 h^15^.

**Fig. 4:**
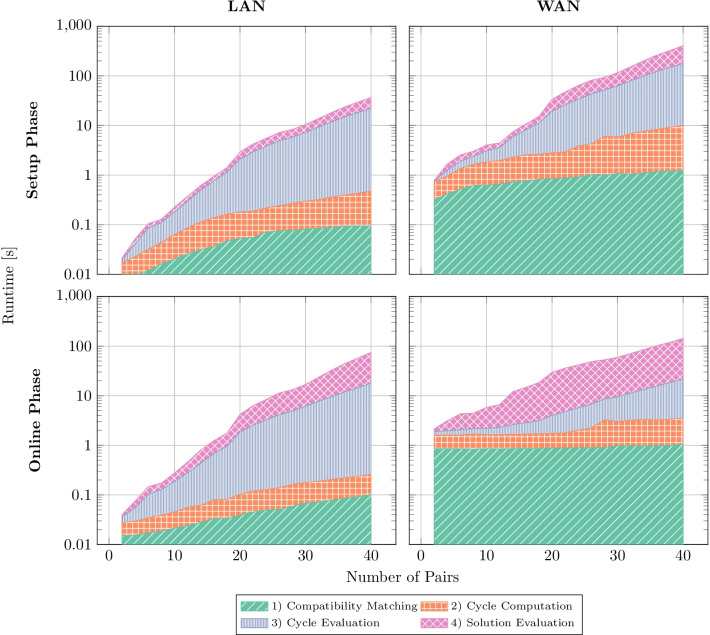
Runtime of SPIKE for $L=2$ separated by algorithmic parts, protocol phase, and network setting

Figure 4 shows the runtimes of the individual parts of the algorithm ($L=2$). It is clearly visible, that the medical compatibility testing and graph creation, as well as the cycle computation quickly become negligible compared to the runtimes of cycle evaluation and the evaluation of the global solution. The duration of online and offline phases are in the same order of magnitude. By executing the phases separately, a 134% performance increase in the online execution can be achieved, compared to the accumulated runtime (cf. Fig. 3).

### Comparison to state-of-the-art

**Fig. 5:**
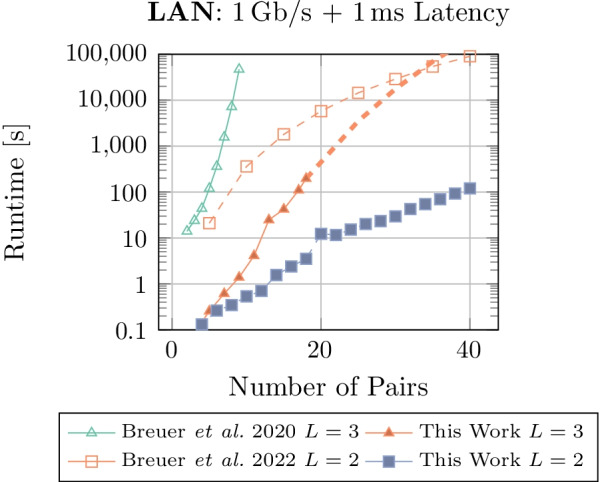
Runtime comparison between this work with cycle lengths $L=2$ and $L=3$, and both Breuer *et al.* 2020 ($L=3$) [9] and Breuer *et al.* 2022 ($L=2$) [10]. All measurements use a LAN network setting with 1Gb/s bandwidth and 1ms latency. The dashed line shows the extrapolated power function for our algorithm at $L=3$

In Fig. 5, we compare the runtime of our implementation for $L=2$ and $L=3$ with two implementations from Breuer et al. [9, 10]. The first implementation [9] uses a Threshold Homomorphic Encryption scheme and enables to solve the privacy-preserving KEP with arbitrary cycle length, as in SPIKE. The maximum cycle size is set to $L=3$ in their benchmarks. The second one [10] is based on three-party honest majority Shamir’s Secret Sharing using the MP-SPDZ framework and limits its cycle length to $L=2$. The performance data for both implementations is taken from the referenced publications.

Our implementation, as well as the MP-SPDZ based state-of-the-art [10], shows a polynomial-bound power-law graph. The Homomorphic Encryption-based implementation shows clearly an exponential runtime development, increasing rapidly. For 9 pairs, the maximum number of pairs benchmarked in the original publication [9], our implementation achieves a ${29828} \times$ speedup. For $L=2$, our implementation performs ${414} \times$ better than the MP-SPDZ-based implementation [10].

**Fig. 6:**
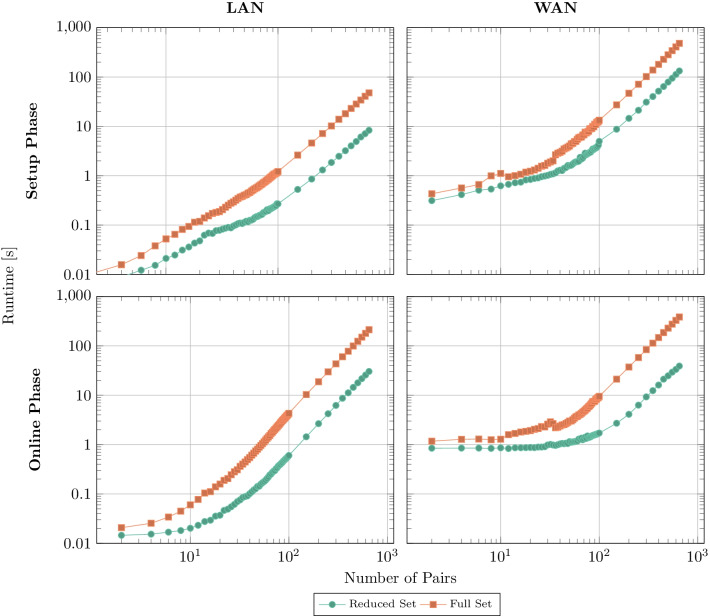
Comparison of the compatibility matching (i.e., compatibility graph generation) performance between the reduced set of criteria (Breuer *et al.* 2020) [9] and the full set of this work for cycle length $L=2$. The runtimes of the remaining algorithmic parts are independent of this choice

To improve the medical quality of the donor-recipient matching, we implemented additional matching criteria, as described in the “Background” section. As we have seen in Fig. 4, the performance impact of the compatibility matching algorithm is negligible compared to the runtime of the remaining algorithmic parts. However, in Fig. 6 we compare the performance difference between the reduced set of medical matching criteria and the full set. For small number of pairs there is a transient phase, where the runtime of the full set rises faster. After this transient phase, both curves assume nearly the same slope. In the plots for the WAN network model, the latency-induced “baseline” runtime can be observed.

A comparison of communication size between SPIKE and [9, 10] for cycle lengths $L=3$ and $L=2$ is included in the appendix in Additional file 1: Tables S16 and S17. For $L=2$ and 40 pairs, SPIKE requires ${40} \times$ less communication than [10]. For $L=3$ and 9 pairs (the maximum number of pairs evaluated by [9]), SPIKE require ${104.1} \times$ less communication than [9].

## Discussion

### Security guarantees

Our privacy-preserving kidney exchange protocol, SPIKE, is implemented using the ABY [61] MPC framework, guaranteeing computational semi-honest security in a two-party setting. An adversary $\mathcal {A}$ can corrupt at most one of the two computing parties. $\mathcal {A}$ is assumed to follow the protocol specification and gets access to all messages of the corrupted party (sent and received), while trying to extract private information. This security model is standard in the privacy research community and protects against two security concerns: (1) inadvertent disclosure of sensitive data and (2) *full* data disclosure in case of a breach in one of the parties (in comparison to a centralized computation). The latter concern is a detriment of all centralized or trusted third party based approaches. This coupled with complex legal barriers are the driving forces behind the German Medical Informatics Initiative’s^16^^,^^17^ decision to promote decentralized data holding and processing. In the outsourcing scenario with two computation parties and an arbitrary number of data sources, both computation parties *must not* collude. However, an arbitrary number of data sources is allowed to collude or behave maliciously, without breaking the security guarantees. Note, that MPC only gives privacy guarantees for the computation, whereas maliciously formed inputs might lead to incorrect outputs. For a “holistic” data privacy perspective, please see [74].

While this adversarial model is not sufficient for all applications, e.g., computations with parties in different jurisdictions [62], it suits our setting, namely the joint computation among large, intra-national or intra-European medical institutions. Both semi-honest behaviour, as well as the non-collusion assumption, can be enforced by legal and regulatory means and build the predominant basis for data protection concepts in (German) federated medical research networks.

Furthermore, several real-world industry projects demonstrate that the non-collusion assumption of MPC is practical. For example, Mozilla Firefox starts to deploy MPC-based privacy-preserving collection for Telemetry data [75, 76] and Bosch is developing a MPC platform for smart homes and anonymous driving [77, 78]. Many more examples can be found in the MPC alliance^18^ which is a consortium of industry peers working on MPC. Even in the German medical informatics realm employed MPC solutions are able to work in a (non-colluding) two-party setting [79].

For a full description of the cryptographic assumptions and guarantees inherited by the primitives used in ABY, we refer to the respective section in the Appendix and the original ABY publication [61].

While all data, including the association to the various data sources are considered to be private data and are protected by the aforementioned guarantees, we consider the *number* of donor-recipient pairs, as well as the maximum number of cycles in the graph, as public information. This choice has important performance impacts, however, if the numbers of pairs are to be considered private as well, the real numbers can be hidden by padding each input array to a fixed length with dummy entries.

### Real-world deployability

This work introduces a protocol for finding a solution for the kidney exchange problem in a privacy-preserving fashion. As demonstrated in the performance benchmarks and the security discussion, it meets all initially determined requirements for a secure privacy-preserving solution to the KEP w.r.t privacy, efficiency, decentralization, and adaptability for medical experts.

Concretely, it enables real-world periodic batch-processing for a significantly larger number of donor-recipient pairs and a practical cycle length of $L=2$ and $L=3$ compared to previous work [9, 10], even in residential network settings. This allows even residential nephrology experts to participate in kidney exchanges, hence, providing a better medical care for their patients. However, SPIKE requires a significant amount of communication, thus, it is not yet ready for the usage of metered or cell data connections in a two-party computation protocol, which might be an interesting direction for future work. In contrast, SPIKE is already practical for an outsourcing scenario, where mobile clients secret share their data of 100 donor-recipient pairs among two non-colluding servers or cloud entities. But we also point out that larger sets of, e.g., 300 pairs are not practical yet due to the, although improved, but still limited scalability of our protocol. To run SPIKE on even larger datasets, two strategies are possible: (1) reducing the interval of calculation, hence, effectively reducing the participating pairs, or (2) partitioning on less sensitive features, such as “blood type” and running the computation on smaller data chunks in parallel. The first approach, however, results in a smaller set of pairs considered in the matching while the second change likely increases the number of mismatches that will not pass the final check by medical experts.

By using state-of-the-art provably secure cryptographic techniques, the privacy of sensitive medical information of donors and recipients is fully protected by clearly defined hardness assumptions of mathematical problems. Furthermore, by pursuing a completely decentralized approach without a trusted third party, the risk of data leakages in case of a data security incident at one participating facility is significantly reduced, compared to a breach in a central computation node or repository. This is especially important for quasi-identifying medical fields^19^. Often times, quasi-identifiers are not anonymizable, as they loose too much utility in the process. Hence, secure decentral storage and processing of non-anonymized data is increasingly important especially considering current efforts of simplified international data usage, such as the proposed European Health Data Space (EHDS) [80].

Allowing medical professionals to choose many parameters of the algorithm to adapt to new evidence-based guidelines or specific situational constraints ensures flexibility and maintainability for future application. The compatibility matching algorithm is configurable by choosing the considered HLA, as well as the weights of the chosen medical factors. This explicitly allows the deactivation of chosen comparisons. Due to the clear architecture boundaries in the open source implementation, additional checks and criteria can easily be included. Many hierarchically ordered optimization goals employed in current KEP solutions [7] can be included in SPIKE via a more involved weight calculation. One open research question is to quantify a possible transplantation success rate difference between globally optimal KEP solvers and our locally optimal solution. We argue, that the medical uncertainty, that can not easily be evaluated via algorithms, e.g., number and positions of renal arteries [81], might be larger than the uncertainty introduced by our local solution. To answer this highly relevant question a cross-examination of followed-up real world kidney-exchanges would be required and is left as future work.

While meeting all formal requirements, SPIKE falls short in two aspects: First, we observe a high memory consumption during the computation. This is expected, as this protocol was optimized for runtime performance. The reason is that hardware costs are typically not a prohibiting factor for meeting data protection regulations. Note that we use only standard hardware for our benchmarks. For a real-world deployment, it is realistic to assume a deployment on servers with significantly higher capacity. Thus, we argue that this aspect does not jeopardize the adoption in the intended use cases. However, improvements in this regard are still desirable. For example, developing internal batch processing of graph clusters and the employment of space-optimized data structures might be worthwhile opportunities for further research. An interesting direction for future work can be to explore the compatibility with recent advances in MPC-based graph analysis for breadth-first search [82] scaling linearly in the number of vertices. Second, the developed software components are research artifacts and fulfil a prototypical function. For real-world adoption the implementation of widespread medical standards, e.g., HL7 FHIR R4^20^, audit- and authentication capabilities, integration in medical research pipelines, creation of deployment packages, and lastly full (legal) documentation must be pursued. This is, however, not in the scope of this work.

## Conclusion

In this work, we introduced SPIKE, the currently most efficient privacy-preserving Kidney Exchange Problem (KEP) protocol. Using provably secure cryptographic techniques, SPIKE provides highest data protection guarantees for patients’ sensitive medical data without relying on a trusted third party, while allowing a decentralized computation of a locally optimal solution to the kidney exchange problem. In the absence of privacy-preserving Integer Linear Programming (ILP) solving algorithms, we implement approximate, adaptable medical compatibility matching algorithms, giving medical professionals the flexibility to accommodate updated guidelines and the specific situational constraints.

Our optimized protocols achieve a ${30000}\times$ and ${400}\times$ speedup compared to the current state-of-the-art [9, 10] for cycle lengths of $L=3$ and $L=2$, respectively. With a total runtime of under 4min for 40 pairs at $L=2$ and around 1 h for 25 pairs at $L=3$, we demonstrate sufficient performance for deploying it for some real-world applications.

However, we note that kidney exchange programs typically consider up to 300 pairs per run [83] which is not yet feasible for SPIKE since our protocol does not scale sufficiently well in the number of participating donor-recipient pairs leading to unsatisfactory runtimes beyond 170 pairs (for $L=2$). Additionally, memory usage is another aspect that needs more future work. To summarize, SPIKE is not yet a routine solution ready for deployment for large scale kidney exchange programs, however, it offers the most efficient state-of-the-art solution to the problem. In this sense, it makes an important contribution towards moving into the direction of practical large-scale privacy-preserving solutions.

We also hope that the advancements in privacy protection and application performance will already allow more medical facilities to participate in kidney exchanges on a smaller scale, thus increasing the recipients’ chances for timely and potentially live-saving surgery.

## Supplementary information

**Additional file 1.** Appendix: Supplementary tables.

## Data Availability

Our code and the datasets analyzed are available here: https://encrypto.de/code/PPKE.

## Abbreviations

MPC: Secure multi-party computation
HE: Homomorphic encryption
SSS: Shamir’s secret sharing
$\small \mathcal {Y}$: Yao’s garbled circuits
$\small \mathcal {B}$: Boolean secret sharing
$\small \mathcal {A}$: Arithmetic secret sharing
PPKE: Privacy-preserving kidney exchange
KEP: Kidney exchange problem
HLA: Human leukocyte antigens

## Footnotes

1 For example, France and Sweden only accept a cycle Length of $L=2$, while Spain, the Netherlands, and the United Kingdom accept longer cycles but prefer $L=2$. For details see [8] and [7].

2 This cycle still requires a final check by medical experts.

3 https://www.gnu.org/licenses/lgpl-3.0

4 As mentioned above, the communication of MPC protocols generally scales quadratically in the number of parties, thus, more parties significantly increase communication cost. When considering full threshold security, runtimes significantly increase as well. The state-of-the-art MPC framework MOTION [60] which offers full threshold security provides several benchmarks comparing to the two-party MPC framework ABY [61]. In a LAN setting, it takes ABY less than 0.1 seconds (online runtime) to securely compute an AES-128 circuit with two parties while MOTION requires at least twice (resp. four times) the time with three (resp. five) parties.

5 For examples see [63] and [64] (in German language).

6 Note that our protocols in Section “Methods” can also be instantiated with other MPC frameworks. For example, an instantiation with MP-SPDZ would also enable the switch to a malicious security model but at the costs of significantly reduced efficiency [71]. However, considering the security and efficiency requirements discussed in the Subsections “Outsourcing Data-Model” and “Security Model”, 2-party MPC with the ABY framework offers the best trade-off.

7 $$A \vee B = 1 \oplus ((1 \oplus A) \wedge (1 \oplus B))$$

8 As discussed in the Related Work, we recommend 2 to 3 to foster robustness.

9 Even though exhibiting a cubic runtime complexity, this part’s performance is negligible compared to the following parts (cf. Fig. 4), hence, an optimization is not vital.

10 Cycle (A, B, C) and cycle (B, C, A) are duplicates, but cycle (C, B, A) is not.

11 Exceptions are fully connected and unconnected graphs, as well as for $|\mathsf {cycles}|=1$ at pathological graph topologies. The first topologies have no security implication whatsoever and the later can, e.g., be easily avoided by introducing a check ensuring that the output is only revealed when more cycles have been found.

12 Calculation of a global solution is provably a $\mathcal{NP}\mathcal{}$-hard problem [73].

13 https://dfn.de/

14 https://man7.org/linux/man-pages/man8/tc.8.html

15 Based on a power function $f(x)=a\cdot x^b + c$ fit with the parameters $a = {0.003563}, b = {4.673}$ and $c = {1005}$ giving the runtime in milliseconds.

16 https://www.medizininformatik-initiative.de/

17 The German Medical Informatics Initiative is the federal research initiative to enable medical data sharing and secondary use between all university medical centers in Germany.

18 https://www.mpcalliance.org/

19 Quasi-Identifier are groups of fields, that do not include the traditional fields of identifying data, such as names and birth dates. Nevertheless, the combination of fields in a quasi-identifier is rare enough to identify individual patients.

20 https://www.hl7.org/fhir/R4/
