# Supplementary material for: SPIKE: secure and private investigation of the kidney exchange problem
Source: BMC Med Inform Decis Mak. 2022 Sep 22;22:253. doi: 10.1186/s12911-022-01994-4 (PMC9502669; doi:10.1186/s12911-022-01994-4)
Supplement: Supplementary file 1 — Additional file 1. Appendix: Supplementary tables. [file 12911_2022_1994_MOESM1_ESM.pdf]

Appendix

Additional MPC Subprotocols

This part of the Appendix contains additional protocols of our MPC-based PPKE solution, SPIKE, that are similar to the ones presented in the main part and, thus, referred to the appendix.

Additional Protocols for the Compatibility Matching

This subsection presents additional subprotocols for the compatibility matching phase.

Supplementary Table 1 $\texttt{evalHLA}(\langle \mathsf{hla_d} \rangle^{\mathcal{B}}$: vector, $\langle \mathsf{hla_r} \rangle^{\mathcal{B}}$: vector) $\rightarrow$ int

1: $\langle \mathsf{mm} \rangle^{\mathcal{B}} \leftarrow \{\langle 0 \rangle^{\mathcal{B}}\}^{|\mathsf{HLA}|}$
2: **for** $i = 0, \ldots, |\mathsf{HLA}| - 1$ **do**
3: $\quad \langle \mathsf{mm} \rangle^{\mathcal{B}} \leftarrow \langle \mathsf{hla_d} \rangle^{\mathcal{B}}[i] \oplus \langle \mathsf{hla_r} \rangle^{\mathcal{B}}$ ▷ SIMD
4: **end for**
5: $\langle \mathsf{sum} \rangle^{\mathcal{B}} \leftarrow \texttt{HammingW}(\{\langle 0 \rangle^{\mathcal{B}}\}^{|\mathsf{HLA}|}, \langle \mathsf{mm} \rangle^{\mathcal{B}})$
6: $\langle \mathsf{c} \rangle^{\mathcal{B}} \leftarrow \langle \mathsf{sum} \rangle^{\mathcal{B}} < \langle 5 \rangle^{\mathcal{B}}$
7: $\langle \mathsf{b} \rangle^{\mathcal{B}} \leftarrow \langle \mathsf{sum} \rangle^{\mathcal{B}} < \langle 3 \rangle^{\mathcal{B}}$
8: $\langle \mathsf{a} \rangle^{\mathcal{B}} \leftarrow \langle \mathsf{sum} \rangle^{\mathcal{B}} == \langle 0 \rangle^{\mathcal{B}}$
9: **return** $\langle \mathsf{a} \rangle^{\mathcal{B}}$ **?** $\langle \mathsf{A} \rangle^{\mathcal{B}} (\langle \mathsf{b} \rangle^{\mathcal{B}}$ **?** $\langle \mathsf{B} \rangle^{\mathcal{B}}$ **:** $(\langle \mathsf{c} \rangle^{\mathcal{B}}$ **?** $\langle \mathsf{C} \rangle^{\mathcal{B}}$ **:** $\langle 0 \rangle^{\mathcal{B}}))$

HLA Antigen Comparison

In Supplementary Table 1, we compare the HLA antigens of the potential recipient and donor and determine the number of HLA mismatches. It takes two vectors $\mathsf{hla_d}$ and $\mathsf{hla_r}$ with the HLA antigens of the donor and recipient respectively as input. The number of |HLA| is public, as it is a fixed value. The vector mm indicates the HLA mismatches of the donor and the recipient. A mismatch occurs if either donor or recipient has a HLA antigen that the other does not have (cf. Line 3). For enhanced efficiency, we parallelize the comparison as SIMD operation, such that the vector mm is computed in a single step. Afterwards, the number of HLA mismatches is determined with a Hamming Weight Circuit (cf. Line 5). Based on the number of mismatches, the subprotocol outputs an indicator for the quality of the pairing w.r.t. the HLA antigens: Class A is an optimal fit with no mismatches, class B is a good fit, and class C is an acceptable fit with 3-4 mismatches (cf. Lines 6-9).

MPC Cost

Line 3 in Supplementary Table 1 evaluates |HLA|×`XOR` gates (as SIMD). Line 5 evaluates one Hamming Distance circuit. Lines 6-9 contain three comparison and three `MUX` gates. Thus, the circuit's multiplicative depth is 7, which is determined by the number of `AND` gates on the longest path. Naively, using Yao's Garbled Circuits ($\mathcal{Y}$) seems to be most efficient. However, considering that Table 4 is done in $\mathcal{B}$ sharing, the conversion cost outweigh the benefits of using $\mathcal{Y}$ instead of $\mathcal{B}$, thus, $\mathcal{B}$ is used here as well.

Supplementary Table 2 $\texttt{evalABO}(\langle \mathsf{bg_d} \rangle^{\mathcal{B}}$: vector, $\langle \mathsf{bg_r} \rangle^{\mathcal{B}}$: vector) $\rightarrow$ int

1: $\langle \mathsf{a} \rangle^{\mathcal{B}} \leftarrow \neg\Big((\langle \mathsf{bg_r} \rangle^{\mathcal{B}}[0] \oplus \langle \mathsf{bg_d} \rangle^{\mathcal{B}}[0]) \vee (\langle \mathsf{bg_r} \rangle^{\mathcal{B}}[1] \oplus \langle \mathsf{bg_d} \rangle^{\mathcal{B}}[1]) \Big)$
2: $\langle \mathsf{b} \rangle^{\mathcal{B}} \leftarrow (\langle \mathsf{bg_r} \rangle^{\mathcal{B}}[1] \wedge \neg \langle \mathsf{bg_d} \rangle^{\mathcal{B}}[0]) \vee (\langle \mathsf{bg_r} \rangle^{\mathcal{B}}[0] \wedge \neg \langle \mathsf{bg_d} \rangle^{\mathcal{B}}[1])$
3: $\langle \mathsf{v} \rangle^{\mathcal{B}} \leftarrow \langle \mathsf{a} \rangle^{\mathcal{B}} \vee \langle \mathsf{b} \rangle^{\mathcal{B}}$
4: **return** $\langle \mathsf{v} \rangle^{\mathcal{B}}$ **?** $\langle \mathsf{best_{age}} \rangle^{\mathcal{B}}$ **:** $\langle 0 \rangle^{\mathcal{B}}$

ABO blood group comparison

Supplementary Table 2 contains the privacy-preserving evaluation of the compatibility of ABO blood groups of a donor and a recipient. It takes two two-bit vectors as input: $bg_d \in \{0,1\}^2$ is the blood group of the donor and $bg_r \in \{0,1\}^2$ is the blood group of the recipient. The blood group encoding is shown in Supplementary Table 3. Lines 1-2 ensure that the blood group of recipient and donor are compatible, i.e., they have to be either equal, $bg_r[1] > bg_d[0]$, or $bg_r[0] > bg_d[1]$ (cf. Table 2).

Supplementary Table 3 Encoding of the different blood groups.

Encoding	Blood Group
00	O
01	A
10	B
11	AB

MPC Cost

Here, we evaluate 14 XOR gates and five AND gates in total per donor/recipient pair. As `XOR` gates can be locally evaluated, they are "for free". Therefore, the `AND` gates and circuit depth determine, which MPC protocol is most efficient. $\mathcal{B}$ is slightly more efficient than $\mathcal{Y}$ since the circuit depth is smaller than the number of total AND gates.

Supplementary Table 4 $\texttt{evalAge}(\langle \mathsf{a_d} \rangle^{\mathcal{B}}$: int, $\langle \mathsf{a_r} \rangle^{\mathcal{B}}$: int) $\rightarrow$ int

1: $\langle \mathsf{eq} \rangle^{\mathcal{B}} \leftarrow \langle \mathsf{a_d} \rangle^{\mathcal{B}} == \langle \mathsf{a_r} \rangle^{\mathcal{B}}$
2: $\langle \mathsf{yg} \rangle^{\mathcal{B}} \leftarrow \neg \langle \mathsf{a_d} \rangle^{\mathcal{B}} \wedge \langle \mathsf{a_r} \rangle^{\mathcal{B}}$
3: **return** $\langle \mathsf{yg} \rangle^{\mathcal{B}}$ **?** $(\langle \mathsf{eq} \rangle^{\mathcal{B}}$ **?** $\langle \mathsf{A} \rangle^{\mathcal{B}}$ **:** $\langle \mathsf{B} \rangle^{\mathcal{B}})$ **:** $(\langle \mathsf{eq} \rangle^{\mathcal{B}}$ **?** $\langle \mathsf{A} \rangle^{\mathcal{B}}$ **:** $\langle 0 \rangle^{\mathcal{B}})$

Age Comparison

Supplementary Table 4 evaluates the compatibility of a donor and recipient based on their age group. It takes the age group of the donor $\langle a_d \rangle^B$ and the age group of the recipient $\langle a_r \rangle^B$ as input. Line 1 checks if they are in the same age group and Line 2 evaluates whether the donor is in a younger age group than the recipient. Afterwards, we compute the respective weight of this donor and recipient constellation. Similarly, as in Supplementary Table 1, class A indicates an optimal match, class B a good match, and Eq denotes that recipient and donor are in the same age group.

MPC Cost

Supplementary Table 4 contains one comparison, one inversion, one `AND` gate, and three `MUX` gates. As Line 1 and Line 2 are independent, similarly as the two `MUX` gates in Line 3, the circuit depth is 3. Thus, this subprotocol is slightly more efficient in $\mathcal{B}$ than in $\mathcal{Y}$.

Supplementary Table 5 $\texttt{evalSex}(\langle \mathsf{s_d} \rangle^{\mathcal{B}}$: int, $\langle \mathsf{s_r} \rangle^{\mathcal{B}}$: int) $\rightarrow$ int

1: $\langle \mathsf{eq} \rangle^{\mathcal{B}} \leftarrow \langle \mathsf{s_d} \rangle^{\mathcal{B}} == \langle \mathsf{s_r} \rangle^{\mathcal{B}}$
2: $\langle \mathsf{fdmr} \rangle^{\mathcal{B}} \leftarrow \langle \mathsf{s_d} \rangle^{\mathcal{B}} \wedge \neg \langle \mathsf{s_r} \rangle^{\mathcal{B}}$
3: **return** $\langle \mathsf{fdmr} \rangle^{\mathcal{B}}$ **?** $(\langle \mathsf{eq} \rangle^{\mathcal{B}}$ **?** $\langle \mathsf{A} \rangle^{\mathcal{B}}$ **:** $\langle 0 \rangle^{\mathcal{B}})$ **:** $(\langle \mathsf{eq} \rangle^{\mathcal{B}}$ **?** $\langle \mathsf{A} \rangle^{\mathcal{B}}$ **:** $\langle \mathsf{B} \rangle^{\mathcal{B}})$

Sex Comparison

Supplementary Table 5 evaluates the compatibility of a donor and recipient based on their sex. It takes two secret shares $\langle s_d \rangle^B$ and $\langle s_r \rangle^B$ as input, which represent the sex of the donor and recipient, respectively. In Line 1, the subprotocol determines if the pair shares the same sex. Line 2 checks whether the donor is female and the recipient male. As final step, the output weight of this donor and recipient constellation is computed, i.e., the optimal combination ("Class A") with equal sex receives the highest weight, while a female donor and a male recipient are assigned the lowest weight (0).

MPC Cost

Supplementary Table 5 evaluates one comparison, one inversion, one `AND`, and three `MUX` gates. As Line 1 and Line 2, as well as two of the `MUX` gates in Line 3, are independent, we have a circuit depth of 3. Thus, Supplementary Table 5 is slightly more efficient in $\mathcal{B}$ than in $\mathcal{Y}$.

Weight Comparison

Supplementary Table 6 evaluates the compatibility of a donor and recipient based on their weight. It takes two secret shares as input: $\langle w_d \rangle^B$ and

Supplementary Table 6 $\texttt{evalWeight}(\langle \mathsf{w_d} \rangle^{\mathcal{B}}$: int, $\langle \mathsf{w_r} \rangle^{\mathcal{B}}$: int) $\rightarrow$ int

1: **return** $\langle \mathsf{w_d} \rangle^{\mathcal{B}} < \langle \mathsf{w_r} \rangle^{\mathcal{B}}$ **?** $\langle 0 \rangle^{\mathcal{B}}$ **:** $\langle \mathsf{A} \rangle^{\mathcal{B}}$

$\langle w_r \rangle^B$, which represent the weight of the donor and recipient, respectively. If the donor weighs less than the recipient, it returns a secret shared 0, otherwise, it indicates a good fit (i.e., class "A" w.r.t. criteria weight).

MPC Cost
We evaluate only one comparison gate. As the evaluation of a single comparison is more efficient in $\mathcal{Y}$ than in $\mathcal{B}$ [1], $\mathcal{Y}$ would be more efficient. However, the conversion cost outweigh this benefit, which is why $\mathcal{B}$ is used for this subprotocol as in the previous comparison protocols.

Additional Protocols for the Cycle Computation

Supplementary Table 7 $\texttt{removeWeights}(\langle \mathsf{compG} \rangle^{\mathcal{B}}$: matrix) $\rightarrow$ matrix

1: $\langle \mathsf{uG} \rangle^{\mathcal{A}} \leftarrow$ matrix $\in \langle 0 \rangle^{\mathcal{A}^{|\mathsf{pairs}|}}$
2: **for** $i = 0, \ldots, |\mathsf{pairs}| - 1$ **do**
3: **for** $j = 0, \ldots, |\mathsf{pairs}| - 1$ **do**
4: $\langle \mathsf{uG} \rangle^{\mathcal{A}}[i][j] \leftarrow$ $\mathtt{b2a}(\langle \mathsf{compG} \rangle^{\mathcal{B}}[i][j] > \langle 0 \rangle^{\mathcal{B}}$ **?** $\langle 1 \rangle^{\mathcal{B}}$ **:** $\langle 0 \rangle^{\mathcal{B}}$
5: **end for**
6: **end for**
7: **return** $\langle \mathsf{uG} \rangle^{\mathcal{A}}$

Weight Removal
In Supplementary Table 7, we compute the unweighted compatibility graph, which is used for determining the number of cycles for the desired cycle length. It takes the weighted compatibility graph *compG* as input. The number of donor-recipient pairs |pairs| is public. In Line 4, we remove the edge weights: If it is greater than 0, it is set to 1, otherwise to 0. As preparation for later processing, a conversion to $\mathcal{A}$ is done.

MPC Cost
The subprotocol shown in Supplementary Table 7 evaluates $|\mathsf{pairs}|^2$ comparisons, `MUX` gates, and conversions. The comparisons and `MUX` gates are independent, which results in a circuit depth of 2. Due to the total number of `AND` gates, which is $2 \times |\mathsf{pairs}|$, this subprotocol is most efficient in $\mathcal{B}$.

kNN Sort Protocol
Our next MPC subprotocol shown in Supplementary Table 8 is a k-nearest neighbor sort (a slightly adapted version of the protocol in [2]) that identifies the k most robust cycles (i.e., with the highest likelihood to result in successful transplantations).
It takes a secret shared vector of tuples cyclesSet with exchange cycles and their respective weights and k as input. The length of cycles cLen is a public parameter. First, the subprotocol iterates over all cycles in |cyclesSet| to perform an insertion sort. Each cycle and the respective weight are added to sortedC and sortedW if its weight is one of the k highest weights (cf. Lines 11 to 27). Thus, the final sortedW and sortedC are sorted in decreasing order with respect to the weights of cycles.

MPC Cost
This subprotocol evaluates $|\mathsf{cyclesSet}| \times k$ comparisons and $|\mathsf{cyclesSet}| \times k \times (1 + \mathsf{cLen})$ `MUX` gates. It is most efficient in $\mathcal{Y}$ due to depth of the circuit determined by the number of `AND` gates.

Duplicate Removal
Supplementary Table 9 removes all duplicated exchange cycles and outputs the remaining $|\mathsf{unique}| = \lfloor \frac{|\mathsf{cycles}|}{\mathsf{cLen}} \rfloor$ cycles.
It takes a secret shared vector of tuples sortedCycles as input, which contains cycles and weights sorted according to the respective weights (i.e., the output by Supplementary Table 8). The number of existing cycles

Supplementary Table 8 $\texttt{kNNSort}(\langle \mathsf{cyclesSet} \rangle^{\mathcal{Y}}$: vector of tuples, k: int) $\rightarrow$ vector of cycles

1: $\langle \mathsf{sortedW} \rangle^{\mathcal{Y}} \leftarrow \emptyset$
2: $\langle \mathsf{sortedC} \rangle^{\mathcal{Y}} \leftarrow \emptyset$
3: **for** $i = 0, \ldots, k$ **do**
4: $\langle \mathsf{sortedW} \rangle^{\mathcal{Y}}.\mathtt{append}(\langle 0 \rangle^{\mathcal{Y}})$
5: $\langle \mathsf{vertices} \rangle^{\mathcal{Y}} \leftarrow \emptyset$
6: **for** $j = 0, \ldots, \mathsf{cLen} - 1$ **do**
7: $\langle \mathsf{vertices} \rangle^{\mathcal{Y}}.\mathtt{append}(|\langle \mathsf{pairs} \rangle^{\mathcal{Y}}|)$
8: **end for**
9: $\langle \mathsf{sortedC} \rangle^{\mathcal{Y}}.\mathtt{append}(\langle \mathsf{vertices} \rangle^{\mathcal{Y}})$
10: **end for**
11: **for** $i = 0, \ldots, |\mathsf{cyclesSet}| - 1$ **do**
12: $\langle \mathsf{sortedW} \rangle^{\mathcal{Y}}[k] \leftarrow \langle \mathsf{cyclesSet} \rangle^{\mathcal{Y}}[i][0]$
13: $\langle \mathsf{sortedC} \rangle^{\mathcal{Y}}[k] \leftarrow \langle \mathsf{cyclesSet} \rangle^{\mathcal{Y}}[i][1]$
14: **for** $j = 0, \ldots, k - 1$ **do**
15: $\langle \mathsf{sel} \rangle^{\mathcal{Y}} \leftarrow \langle \mathsf{sortedW} \rangle^{\mathcal{Y}}[j] > \langle \mathsf{sortedW} \rangle^{\mathcal{Y}}[j-1]$
16: $\langle \mathsf{tmp1} \rangle^{\mathcal{Y}} \leftarrow \langle \mathsf{sortedW} \rangle^{\mathcal{Y}}[j]$
17: $\langle \mathsf{tmp2} \rangle^{\mathcal{Y}} \leftarrow \langle \mathsf{sortedW} \rangle^{\mathcal{Y}}[j-1]$
18: $\langle \mathsf{sortedW} \rangle^{\mathcal{Y}}[j] \leftarrow \langle \mathsf{sel} \rangle^{\mathcal{Y}}$ **?** $\langle \mathsf{tmp2} \rangle^{\mathcal{Y}}$ **:** $\langle \mathsf{tmp1} \rangle^{\mathcal{Y}}$
19: $\langle \mathsf{sortedW} \rangle^{\mathcal{Y}}[j-1] \leftarrow \langle \mathsf{sel} \rangle^{\mathcal{Y}}$ **?** $\langle \mathsf{tmp1} \rangle^{\mathcal{Y}}$ **:** $\langle \mathsf{tmp2} \rangle^{\mathcal{Y}}$
20: **for** $l = 0, \ldots, \mathsf{cLen} - 1$ **do**
21: $\langle \mathsf{tmp1} \rangle^{\mathcal{Y}} \leftarrow \langle \mathsf{sortedC} \rangle^{\mathcal{Y}}[j][l]$
22: $\langle \mathsf{tmp2} \rangle^{\mathcal{Y}} \leftarrow \langle \mathsf{sortedC} \rangle^{\mathcal{Y}}[j-1][l]$
23: $\langle \mathsf{sortedC} \rangle^{\mathcal{Y}}[j][l] \leftarrow \langle \mathsf{sel} \rangle^{\mathcal{Y}}$ **?** $\langle \mathsf{tmp2} \rangle^{\mathcal{Y}}$ **:** $\langle \mathsf{tmp1} \rangle^{\mathcal{Y}}$
24: $\langle \mathsf{sortedC} \rangle^{\mathcal{Y}}[j-1][l] \leftarrow$ $\langle \mathsf{sel} \rangle^{\mathcal{Y}}$ **?** $\langle \mathsf{tmp1} \rangle^{\mathcal{Y}}$ **:** $\langle \mathsf{tmp2} \rangle^{\mathcal{Y}}$
25: **end for**
26: **end for**
27: **end for**
28: $\langle \mathsf{result} \rangle^{\mathcal{Y}} \leftarrow \emptyset$
29: **for** $i = 0, \ldots, |\mathsf{cycles}| - 1$ **do**
30: $\langle \mathsf{result} \rangle^{\mathcal{Y}}.\mathtt{append}(\mathsf{tuple}(\langle \mathsf{sortedW} \rangle^{\mathcal{Y}}[i], \langle \mathsf{sortedC} \rangle^{\mathcal{Y}}[i]))$
31: **end for**
32: **return** $\langle \mathsf{result} \rangle^{\mathcal{Y}}$

|cycles|, the number of unique cycles |unique|, and the cycle length cLen are public parameters. For each cycle $c1$ in sortedCycles, it is checked if it is equal to any other cycle $c2$ (cf. Lines 6 to 13). If this is the case, its weight is set to 0 (cf. Line 15). To each equality, it is evaluated if the vertex of $c1$ at index l and the vertex of $c2$ at index $(l+k) \mod \text{cLen}$ are identical (cf. Line 9). With Supplementary Table 8, sortedCycles is sorted and only the |unique| cycles with the highest weight are returned. The number of unique cycles is $|\text{unique}| = \lfloor \frac{|\text{cycles}|}{\text{cLen}} \rfloor$.

MPC Cost

Supplementary Table 9 has $|\text{cycles}| \times \sum_{i=0}^{|\text{cycles}|} (\text{cLen} \times (\text{cLen}-1))$ comparisons and AND gates, $|\text{cycles}| \times \sum_{i=0}^{|\text{cycles}|} (\text{cLen}-1)$ OR gates, |cycles| MUX gates. Including Supplementary Table 8, this results in $|\text{cycles}| \times |\text{unique}|$ comparison and MUX gates, and an additional $|\text{cycles}| \times |\text{unique}| \times (1+\text{cLen})$ MUX gates. This subprotocol is most efficient in $\mathcal{Y}$ due to the depth of the circuit created by AND gates.

Supplementary Table 9 `removeDuplicates`($\langle\text{sortedCycles}\rangle^{\mathcal{Y}}$: vector of tuples) $\rightarrow$ vector of cycles

1: **for** $i = 0, \ldots, |\text{cycles}| - 1$ **do**
2: $\quad \langle\text{c1}\rangle^{\mathcal{Y}} \leftarrow \langle\text{sortedCycles}\rangle^{\mathcal{Y}}[i][1]$
3: $\quad \langle\text{combDup}\rangle^{\mathcal{Y}} \leftarrow \langle 0\rangle^{\mathcal{Y}}$
4: $\quad$ **for** $j = 0 : i$ **do**
5: $\quad\quad \langle\text{c2}\rangle^{\mathcal{Y}} \leftarrow \langle\text{sortedCycles}\rangle^{\mathcal{Y}}[j][1]$
6: $\quad\quad$ **for** $k = 1, \ldots, \text{cLen} - 1$ **do**
7: $\quad\quad\quad \langle\text{duplicate}\rangle^{\mathcal{Y}} \leftarrow \langle 1\rangle^{\mathcal{Y}}$
8: $\quad\quad\quad$ **for** $l = 0, \ldots, \text{cLen} - 1$ **do**
9: $\quad\quad\quad\quad \langle\text{same}\rangle^{\mathcal{Y}} \leftarrow \langle\text{c1}\rangle^{\mathcal{Y}}[l] == \langle\text{c2}\rangle^{\mathcal{Y}}[(l+k) \mod \text{cLen}]$
10: $\quad\quad\quad\quad \langle\text{duplicate}\rangle^{\mathcal{Y}} \leftarrow \langle\text{duplicate}\rangle^{\mathcal{Y}} \wedge \langle\text{same}\rangle^{\mathcal{Y}}$
11: $\quad\quad\quad$ **end for**
12: $\quad\quad\quad \langle\text{combDup}\rangle^{\mathcal{Y}} \leftarrow \langle\text{combDup}\rangle^{\mathcal{Y}} \vee \langle\text{duplicate}\rangle^{\mathcal{Y}}$
13: $\quad\quad$ **end for**
14: $\quad$ **end for**
15: $\quad \langle\text{sortedCycles}\rangle^{\mathcal{Y}}[i][0] \leftarrow \langle\text{isDuplicate}\rangle^{\mathcal{Y}}$ **?** $\langle 0\rangle^{\mathcal{Y}}$ **:** $\langle\text{sortedCycles}\rangle^{\mathcal{Y}}[i][0]$
16: **end for**
17: **return** `kNNSort`($\langle\text{sortedCycles}\rangle^{\mathcal{Y}}, |\text{unique}|$)

Supplementary Table 10 `#TotalCycles()` $\rightarrow$ int

$|\text{allCycles}| \leftarrow |\text{pairs}|$
for $i = 1, \ldots, \text{cLen} - 1$ **do**
$\quad |\text{allCycles}| \leftarrow |\text{allCycles}| \cdot (|\text{pairs}| - i)$
end for
return |allCycles|

Total Number of Cycles

Supplementary Table 10 computes the maximum number of cycles that can exist in the compatibility graph. Each vertex must appear at most once in a cycle, which limits the number of possible cycles. As the numbers of pairs |pairs| and the cycle length cLen are public, computation can be done on plaintext.

Additional Protocols for the Solution Evaluation

Disjoint Cycles

Supplementary Table 11 computes whether a cycle `cCycle` does not join vertices with other cycles of a set of cycles `cycles`. It takes as input the set of secret shared cycles `cycles`, the secret shared cycle `cCycle`, and the number of cycles in `cycles` count. If another cycle shares a vertex with `cCycle`, `disJ` is set to 1 (cf. Line 10). In Line 12, we invert the result for further evaluation.

MPC Cost

In this subprotocol, we evaluate $|\text{cycles}| \times \text{cLen}$ (cf. Line 6). In Line 10, we evaluate $\log_2(\text{cLen})$ OR gates. At the end, we evaluate one XOR gate. As the number of total AND gates is greater than the depth of the circuit, this subprotocol is most efficient in $\mathcal{B}$.

Supplementary Table 11 `disjointSet`($\langle\text{cycles}\rangle^{\mathcal{B}}$: vector of tuples, $\langle\text{cCycle}\rangle^{\mathcal{B}}$: vector, $count$: int) $\rightarrow$ Boolean

1: $\langle\text{disJ}\rangle^{\mathcal{B}} \leftarrow \emptyset$
2: **for** $i = 0, \ldots, \text{count} - 1$ **do**
3: $\quad \langle\text{c}\rangle^{\mathcal{B}} \leftarrow \langle\text{cycles}\rangle^{\mathcal{B}}[i][1]$
4: $\quad$ **for** $j = 0, \ldots, \text{cLen} - 1$ **do**
5: $\quad\quad$ **for** $k = 0, \ldots, \text{cLen} - 1$ **do**
6: $\quad\quad\quad \langle\text{tmp}\rangle^{\mathcal{B}} \leftarrow \langle\text{c}\rangle^{\mathcal{B}}[j] == \langle\text{cCycle}\rangle^{\mathcal{B}}[k]$
7: $\quad\quad\quad \langle\text{disJ}\rangle^{\mathcal{B}}.\text{append}(\langle\text{tmp}\rangle^{\mathcal{B}})$
8: $\quad\quad$ **end for**
9: $\quad$ **end for**
10: $\quad \langle\text{disJ}\rangle^{\mathcal{B}} \leftarrow$ `ORTREE`$(\langle\text{disJ}\rangle^{\mathcal{B}})$
11: **end for**
12: **return** $\neg\langle\text{disJ}\rangle^{\mathcal{B}}[0]$

Supplementary Table 12 `findMaximumSet`($\langle\text{cyclesSets}\rangle^{\mathcal{Y}}$: vector of tuples, $\langle\text{cycleW}\rangle^{\mathcal{Y}}$: vector) $\rightarrow$ tuple

$\langle\text{weights}\rangle^{\mathcal{Y}} \leftarrow \emptyset$
$\langle\text{tmp}\rangle^{\mathcal{Y}} \leftarrow \emptyset$
for $i = 0, 1$ **do**
$\quad \langle\text{weights}\rangle^{\mathcal{Y}}.\text{append}(\langle 0\rangle^{\mathcal{Y}})$
$\quad \langle\text{sets}\rangle^{\mathcal{Y}} \leftarrow \emptyset$
$\quad$ **for** $j = 0, \ldots |\text{unique}| - 1$ **do**
$\quad\quad \langle\text{vertices}\rangle^{\mathcal{Y}} \leftarrow \emptyset$
$\quad\quad$ **for** $j = 0, \ldots \text{cLen} - 1$ **do**
$\quad\quad\quad \langle\text{vertices}\rangle^{\mathcal{Y}}.\text{append}(\langle|\text{pairs}|\rangle^{\mathcal{Y}})$
$\quad\quad$ **end for**
$\quad\quad \langle\text{tmp}\rangle^{\mathcal{Y}}.\text{append}(\langle\text{vertices}\rangle^{\mathcal{Y}})$
$\quad$ **end for**
$\quad \langle\text{sets}\rangle^{\mathcal{Y}}.\text{append}(\langle\text{tmp}\rangle^{\mathcal{Y}})$
end for
for $i = 0, \ldots, |\text{unique}| - 1$ **do**
$\quad \langle\text{weights}\rangle^{\mathcal{Y}}[1] \leftarrow \langle\text{cycleW}\rangle^{\mathcal{Y}}[i]$
$\quad \langle\text{sets}\rangle^{\mathcal{Y}}[1] \leftarrow \langle\text{cycleSets}\rangle^{\mathcal{Y}}[i]$
$\quad \langle\text{sel}\rangle^{\mathcal{Y}} \leftarrow \langle\text{weights}\rangle^{\mathcal{Y}}[1] > \langle\text{weights}\rangle^{\mathcal{Y}}[0]$
$\quad \langle\text{tmp1}\rangle^{\mathcal{Y}} \leftarrow \langle\text{weights}\rangle^{\mathcal{Y}}[1]$
$\quad \langle\text{tmp2}\rangle^{\mathcal{Y}} \leftarrow \langle\text{weights}\rangle^{\mathcal{Y}}[0]$
$\quad \langle\text{weights}\rangle^{\mathcal{Y}}[1] \leftarrow \langle\text{sel}\rangle^{\mathcal{Y}}$ **?** $\langle\text{tmp2}\rangle^{\mathcal{Y}}$ **:** $\langle\text{tmp1}\rangle^{\mathcal{Y}}$
$\quad \langle\text{weights}\rangle^{\mathcal{Y}}[0] \leftarrow \langle\text{sel}\rangle^{\mathcal{Y}}$ **?** $\langle\text{tmp1}\rangle^{\mathcal{Y}}$ **:** $\langle\text{tmp2}\rangle^{\mathcal{Y}}$
$\quad$ **for** $j = 0, \ldots, |\text{unique}| - 1$ **do**
$\quad\quad \langle\text{tmp1}\rangle^{\mathcal{Y}} \leftarrow \langle\text{sets}\rangle^{\mathcal{Y}}[1][j]$
$\quad\quad \langle\text{tmp2}\rangle^{\mathcal{Y}} \leftarrow \langle\text{sets}\rangle^{\mathcal{Y}}[0][j]$
$\quad\quad \langle\text{sets}\rangle^{\mathcal{Y}}[1][j] \leftarrow \langle\text{sel}\rangle^{\mathcal{Y}}$ **?** $\langle\text{tmp2}\rangle^{\mathcal{Y}}$ **:** $\langle\text{tmp1}\rangle^{\mathcal{Y}}$
$\quad\quad \langle\text{sets}\rangle^{\mathcal{Y}}[0][j] \leftarrow \langle\text{sel}\rangle^{\mathcal{Y}}$ **?** $\langle\text{tmp1}\rangle^{\mathcal{Y}}$ **:** $\langle\text{tmp2}\rangle^{\mathcal{Y}}$
$\quad$ **end for**
end for
return $(\langle\text{weights}\rangle^{\mathcal{Y}}[0], \langle\text{sets}\rangle^{\mathcal{Y}}[0])$

Maximum Set

Supplementary Table 12 computes the set of cycles with the highest sum of weights, thus, the set of cycles with the highest probability for successful transplantations. Note that we do not compute the globally optimal solution, but a local optimum.

The subprotocol takes a secret shared vector of tuples cycles and a secret shared vector weights as input. cycles contains all sets of disjoint cycles and weights contains the respective weights of the each set. The number of pairs |pairs| and the number of unique cycles |unique| are public parameters. This subprotocol is a slight variation of Table 8 adapted to here used data structures.

The parameter k is fixed to 1 since we look for the set with the highest weight.

MPC Cost
This protocols evaluates $|\text{unique}|$ comparison and $2|\text{unique}|^2 + 2|\text{unique}|$ `MUX` gates. Due to the large number of `MUX` gates in combination with the number of `AND` gates determining the depth of the circuit, it is most efficient in $\mathcal{Y}$ sharing.

Communication Improvement with ABY2.0
Implementing SPIKE in ABY2.0 [3] can further decrease the communication cost. As the respective protocols were implemented only very recently in MOTION2NX [4], we use ABY [1] in our benchmarks to show practicality and additionally discuss the improvements that can be achieved with ABY2.0 in the following.
ABY2.0's improvements for secure multiplication with two inputs decreases the communication of the first and second part, the compatibility matching and the cycle computation. The improvements to conversions between different sharing types additionally benefit the first and the second part, as these parts contain the most conversions between $\mathcal{B}$ and $\mathcal{A}$. Further, the optimizations for matrix multiplications are beneficial for the second part. Concretely, the communication of the protocol in Table 5 decreases from $3 \times \ell^2 + 24 \times \ell + 2 \times \ell \times \kappa$ bits to $23 \times \ell + \ell \times \kappa$ bits in every iteration of the inner loop (without considering the subprotocols). Similarly, the communication of the protocol in Table 6 can be reduced from $\ell \times (\frac{\ell}{2} + 2 \times \kappa + 4 \times |\text{pairs}|^3 + 1.5)$ bits to $\ell \times (\kappa + 2 \times |\text{pairs}|^3 + 3)$ bits, where ℓ is the bitlength and κ is the security parameter.

ABY Security Assumptions and Guarantees
The ABY MPC framework [1] provides mixed-abstraction building blocks for the creation of highly efficient hybrid-protocol MPC applications in a semi-honest adversary setting. Independent of the specific circuit design, a number of base-OTs are executed in the beginning to setup OT Extensions. The used base-OT primitive [5] guarantees security under the Computational Diffie-Hellman (CDH) hardness assumption. Being closely related to the discrete logarithm problem, this assumption is known to not be quantum resistant. The OT Extension [6, 7] primitive uses fixed-key AES modeled as a random permutation. While still considered secure in a semi-honest setting, theoretical attacks in the active security setting have been demonstrated [8]. Furthermore, ABY relies on the random oracle assumption, implemented by the SHA256 hash function. Similarly, Yao's Garbled Circuits [9] (denoted by $\mathcal{Y}$ in our protocols) directly rely on the random permutation assumptions, while Arithmetic and Boolean Sharing [10] (denoted by $\mathcal{A}/\mathcal{B}$ in our protocols) indirectly rely on those assumptions as a source of randomness. Those protocols can achieve information-theoretical security given a true correlated randomness source.

Detailed Benchmark Results
Supplementary Tables 13 to 15 show the detailed benchmark results for the setup and online phase in all three described network settings (A: LAN + $10\,\mathrm{Gb/s}$, B: LAN + $1\,\mathrm{Gb/s}$, C: WAN) and a cycle length of $L = 2$. Supplementary Tables 18 and 19 show the results for a cycle length of $L = 3$.
Supplementary Table 20, finally, compares the benchmark results of both reduced medical compatibility factor set and the full set. This benchmark was performed in the two network settings A and C, as above.

Supplementary Table 13 Comparison of the communication costs and setup and online runtimes of SPIKE for the three networking configurations A: LAN + $10\,\mathrm{Gb/s}$, B: LAN + $1\,\mathrm{Gb/s}$, C: WAN, and for cycle length $L = 2$. This table contains the aggregated total costs and the individual costs of Phase 1 (Compatibility Matching).

Pairs	Comm. [MiB]		Setup Phase [s]			Online Phase [s]		
Pairs	Setup	Online	A	B	C	A	B	C
Total								
2	0.1	0	0.021	0.021	0.78	0.04	0.039	2.1
4	1.1	0.1	0.052	0.051	1.7	0.075	0.08	3.1
6	3.4	0.3	0.1	0.11	2.5	0.15	0.15	4.3
8	5.6	0.4	0.13	0.17	3	0.17	0.18	4.4
10	12.7	0.8	0.22	0.24	4	0.28	0.29	5.8
12	19.5	1	0.37	0.34	4.4	0.46	0.37	6.6
14	55.8	2.3	0.61	0.68	7.4	0.8	0.88	12
16	95.4	3.4	0.94	1.1	11	1.2	1.3	15
18	159	5.1	1.4	1.6	15	1.8	1.9	18
20	412.1	11.8	2.9	4.9	34	4.2	7.4	30
22	617.8	16.6	4.2	5.2	47	6.3	6.4	36
24	823.3	21.1	5.5	6.7	64	8.4	8.5	42
26	1,104.8	27	7.2	8.7	81	11	11	49
28	1,281.6	30.2	8.3	10	93	13	13	53
30	1,608.3	36.5	10	13	120	17	17	59
32	2,202.9	48.3	14	19	150	24	24	71
34	2,999.7	63.8	18	22	200	33	33	85
36	3,971.7	82.2	24	26	260	44	43	100
38	5,036.2	101.8	29	35	320	57	57	120
40	6,394	126.6	37	45	400	75	75	140
Phase 1: Compatibility Matching								
2	0	0	0.0071	0.0065	0.31	0.015	0.015	0.85
4	0.1	0	0.0093	0.0087	0.42	0.016	0.015	0.85
6	0.2	0	0.012	0.013	0.52	0.017	0.017	0.85
8	0.4	0	0.016	0.016	0.62	0.019	0.018	0.84
10	0.6	0	0.02	0.021	0.62	0.021	0.021	0.85
12	0.8	0	0.026	0.025	0.65	0.024	0.024	0.85
14	1.2	0	0.031	0.032	0.72	0.028	0.028	0.86
16	1.5	0	0.036	0.038	0.75	0.034	0.031	0.86
18	1.9	0	0.047	0.045	0.82	0.033	0.033	0.86
20	2.4	0	0.053	0.054	0.85	0.039	0.039	0.88
22	2.9	0.1	0.055	0.065	0.87	0.045	0.046	0.88
24	3.4	0.1	0.071	0.073	0.9	0.05	0.049	0.89
26	4	0.1	0.075	0.083	1	0.051	0.056	0.9
28	4.6	0.1	0.077	0.085	1	0.059	0.06	0.91
30	5.3	0.1	0.081	0.088	1.1	0.068	0.067	0.97
32	6.1	0.1	0.084	0.09	1.1	0.071	0.069	0.98
34	6.8	0.1	0.087	0.092	1.1	0.079	0.083	0.97
36	7.7	0.1	0.093	0.099	1.2	0.085	0.089	0.97
38	8.6	0.2	0.093	0.11	1.2	0.091	0.098	0.99
40	9.5	0.2	0.094	0.11	1.2	0.1	0.1	1

Supplementary Table 14 Comparison of the communication costs and setup and online runtimes of SPIKE for the three networking configurations A: LAN + $10\,\mathrm{Gb/s}$, B: LAN + $1\,\mathrm{Gb/s}$, C: WAN, and for cycle length $L = 2$. This table contains individual costs of Phase 2 and 3 (Cycle Computation and Evaluation).

Pairs	Comm. [MiB]		Setup Phase [s]			Online Phase [s]		
Pairs	Setup	Online	A	B	C	A	B	C
Phase 2: Cycle Computation								
2	0	0	0.0099	0.0099	0.43	0.013	0.012	0.75
4	0.2	0	0.013	0.013	0.54	0.014	0.014	0.76
6	0.4	0.1	0.02	0.02	0.83	0.017	0.018	0.76
8	0.9	0.1	0.028	0.031	1	0.021	0.021	0.85
10	1.7	0.2	0.043	0.047	1.2	0.024	0.027	0.77
12	2.8	0.3	0.06	0.059	1.3	0.033	0.031	0.79
14	4.3	0.4	0.082	0.087	1.6	0.034	0.04	0.8
16	6.2	0.5	0.1	0.12	1.8	0.047	0.048	0.82
18	8.6	0.7	0.12	0.12	1.8	0.048	0.054	0.84
20	11.6	0.8	0.13	0.14	2	0.063	0.061	0.87
22	15.3	1	0.13	0.17	2	0.075	0.072	0.9
24	19.6	1.2	0.15	0.19	2.9	0.078	0.083	1.1
26	24.6	1.5	0.17	0.23	3.2	0.088	0.1	1.3
28	30.5	1.7	0.19	0.27	5	0.1	0.11	2.4
30	37.2	2	0.22	0.3	4.8	0.11	0.12	2
32	44.8	2.3	0.24	0.35	5.7	0.12	0.14	2.2
34	53.4	2.6	0.27	0.42	6.5	0.13	0.15	2.3
36	63	3	0.3	0.48	7.1	0.13	0.16	2.3
38	73.7	3.4	0.34	0.55	7.9	0.14	0.17	2.3
40	85.6	3.8	0.38	0.63	8.8	0.16	0.18	2.4
Phase 3: Cycle Evaluation								
2	0.1	0	0.0023	0.0027	0.022	0.0086	0.0082	0.3
4	0.7	0.1	0.019	0.02	0.29	0.026	0.03	0.35
6	2.2	0.1	0.054	0.061	0.56	0.068	0.07	0.47
8	3.8	0.2	0.066	0.1	0.74	0.089	0.096	0.48
10	8.6	0.4	0.12	0.13	1.2	0.14	0.16	0.56
12	13.4	0.5	0.21	0.2	1.6	0.22	0.2	0.66
14	35	0.9	0.38	0.41	3.6	0.37	0.43	0.94
16	57.3	1.3	0.62	0.66	5.6	0.6	0.69	1.2
18	90.2	1.8	0.98	1	8.5	0.87	0.95	1.4
20	181.2	3.3	1.9	2.2	17	1.7	1.9	2.3
22	255.2	4.4	2.7	2.9	23	2.4	2.5	3
24	332.8	5.3	3.6	3.8	30	3.1	3.1	3.7
26	431.8	6.5	4.7	4.9	39	4	4	4.5
28	514.4	7.2	5.5	5.7	46	4.7	4.7	5.3
30	635.1	8.4	6.9	7.3	57	6	5.9	6.4
32	815.8	10.4	8.9	12	73	7.5	9.7	8
34	1,037.4	12.8	11	12	92	9.4	9.3	10
36	1,292.4	15.4	15	14	110	12	10	12
38	1,567.8	18	18	19	140	14	14	15
40	1,894.4	21.2	22	23	170	18	18	18

Supplementary Table 15 Comparison of the communication costs and setup and online runtimes of SPIKE for the three networking configurations A: LAN + $10\,\text{Gb/s}$, B: LAN + $1\,\text{Gb/s}$, C: WAN and for cycle length $L = 2$. This table contains individual costs of Phase 4 (Solution Evaluation).

Pairs	Comm. [MiB]		Setup Phase [s]			Online Phase [s]		
Pairs	Setup	Online	A	B	C	A	B	C
Part 4: Solution Evaluation								
2	0	0	0.002	0.0016	0.0071	0.0038	0.0037	0.22
4	0.2	0	0.01	0.01	0.42	0.02	0.021	1.2
6	0.5	0.1	0.019	0.019	0.63	0.044	0.045	2.2
8	0.5	0.1	0.018	0.019	0.62	0.045	0.045	2.2
10	1.8	0.2	0.043	0.041	0.96	0.088	0.088	3.6
12	2.4	0.2	0.078	0.055	0.84	0.18	0.11	4.3
14	15.4	0.9	0.12	0.15	1.5	0.37	0.39	9.4
16	30.4	1.6	0.18	0.26	2.5	0.55	0.55	12
18	58.2	2.6	0.27	0.44	4	0.8	0.84	15
20	216.9	7.6	0.84	2.5	14	2.4	5.4	26
22	344.5	11.2	1.3	2	21	3.8	3.9	31
24	467.5	14.5	1.7	2.7	30	5.2	5.3	36
26	644.4	19	2.3	3.5	38	7.2	7.4	42
28	732.1	21.1	2.5	3.9	41	8.3	8.4	44
30	930.7	26	3.2	4.8	53	11	11	50
32	1,336.2	35.5	4.5	5.7	73	16	14	60
34	1,902.1	48.2	6.4	9.2	100	23	23	72
36	2,608.7	63.7	8.7	12	140	32	32	86
38	3,386.1	80.2	11	16	180	43	43	100
40	4,404.4	101.4	15	21	230	57	57	120

Supplementary Table 16 and Supplementary Table 17 compare the communication size for cycle lengths $L = 2$ and $L = 3$ of this work to [11] and [12], respectively.

Supplementary Table 16 Comparison of total communication cost for cycle length L=2.

Pairs	Communication [MiB]	
Pairs	This Work	Breuer et al. 2022
10	13.4	759
20	423.9	13,311.9
30	1,644.8	71,679.7
40	6,520.6	266,238.8

Supplementary Table 17 Comparison of total communication cost for cycle length L=3.

Pairs	Communication [MiB]	
Pairs	This Work	Breuer et al. 2020
3	0.6	0.4
5	4.3	40
7	20.5	200
9	54.1	5,632
15	2,107.3	–
18	9,644.8	–

Author details

References

1. Demmler, D., Schneider, T., Zohner, M.: ABY - A Framework for Efficient Mixed-Protocol Secure Two-Party Computation. Network and Distributed System Security Symposium(NDSS) (2015)
2. Järvinen, K., Leppäkoski, H., Lohan, E.-S., Richter, P., Schneider, T., Tkachenko, O., Yang, Z.: PILOT: Practical Privacy-Preserving Indoor Localization Using OuTsourcing. In: IEEE European Symposium on Security and Privacy (EuroS&P) (2019)
3. Patra, A., Schneider, T., Suresh, A., Yalame, H.: ABY2.00: Improved Mixed-Protocol Secure Two-Party Computation. In: 30th USENIX Security Symposium (USENIX Security 21), pp. 2165–2182 (2021)
4. Braun, L., Cammarota, R., Schneider, T.: POSTER: A Generic Hybrid 2PC Framework with Application to Private Inference of Unmodified Neural Networks (Extended Abstract). Privacy in Machine Learning Workshop (PriML@NeurIPS'21) (2021)
5. Naor, M., Pinkas, B., Pinkas, B.: Efficient Oblivious Transfer Protocols. In: Proceedings of the Twelfth Annual ACM-SIAM Symposium on Discrete Algorithms (2001)
6. Ishai, Y., Kilian, J., Nissim, K., Petrank, E.: Extending Oblivious Transfers Efficiently. In: Advances in Cryptology - CRYPTO 2003, vol. 2729 (2003)
7. Asharov, G., Lindell, Y., Schneider, T., Zohner, M.: More Efficient Oblivious Transfer Extensions. Journal of Cryptology **30**(3) (2017)
8. Guo, C., Katz, J., Wang, X., Yu, Y.: Efficient and Secure Multiparty Computation from Fixed-Key Block Ciphers. In: 2020 IEEE Symposium on Security and Privacy (SP), pp. 825–841 (2020). IEEE
9. Yao, A.C.-C.: How to Generate and Exchange Secrets. In: 27th Annual Symposium on Foundations of Computer Science (SFCS 1986) (1986)
10. Goldreich, O., Micali, S., Wigderson, A.: How to Play ANY Mental Game. In: Proceedings of the 19th Annual ACM Symposium on Theory of Computing. STOC '87 (1987)
11. Breuer, M., Meyer, U., Wetzel, S., Mühlfeld, A.: A Privacy-Preserving Protocol for the Kidney Exchange Problem. WPES (2020)
12. Breuer, M., Meyer, U., Wetzel, S.: Privacy-Preserving Maximum Matching on General Graphs and its Application to Enable Privacy-Preserving Kidney Exchange. In: ACM Conference on Data and Application Security and Privacy (CODASPY) (2022)

Supplementary Table 18 Comparison of the communication costs and setup and online runtimes of SPIKE for the three networking configurations A: LAN + $10\,\mathrm{Gb/s}$, B: LAN + $1\,\mathrm{Gb/s}$, C: WAN and for cycle length $L = 3$. This table contains the aggregated total costs and the individual costs of Phases 1 and 2 (Compatibility Matching and Cycle Computation).

Pairs	Comm. [MiB]		Setup Phase [s]			Online Phase [s]		
Pairs	Setup	Online	A	B	C	A	B	C
Total								
3	0.5	0.1	0.029	0.028	0.97	0.054	0.056	2.2
5	4	0.3	0.096	0.11	2.2	0.13	0.15	2.9
7	19.7	0.7	0.26	0.27	4.1	0.3	0.34	4.3
9	52.6	1.5	0.63	0.66	7.3	0.63	0.73	5.3
11	182.5	3.3	2	2.1	19	1.9	2	9.4
13	1,215.8	16.3	12	13	110	12	12	34
15	2,084.4	22.8	21	23	180	19	20	45
17	5,428.5	58.1	52	56	440	54	54	93
18	9,537.2	107.6	88	95	740	100	100	150
Phase 1: Compatibility Matching								
3	0.1	0	0.0079	0.0075	0.32	0.015	0.015	0.85
5	0.1	0	0.011	0.01	0.42	0.016	0.016	0.85
7	0.3	0	0.014	0.014	0.52	0.018	0.018	0.85
9	0.5	0	0.019	0.018	0.61	0.019	0.02	0.85
11	0.7	0	0.023	0.024	0.64	0.023	0.023	0.86
13	1	0	0.029	0.029	0.72	0.025	0.024	0.86
15	1.3	0	0.034	0.036	0.74	0.029	0.029	0.86
17	1.7	0	0.041	0.043	0.77	0.035	0.034	0.87
18	1.9	0	0.042	0.049	0.82	0.035	0.034	0.87
Phase 2: Cycle Computation								
3	0.1	0	0.013	0.012	0.54	0.014	0.013	0.76
5	0.4	0	0.018	0.019	0.83	0.016	0.016	0.76
7	1.1	0.1	0.029	0.031	1	0.019	0.019	0.77
9	2.2	0.2	0.052	0.053	1.3	0.024	0.023	0.77
11	3.8	0.3	0.072	0.079	1.5	0.026	0.029	0.78
13	6.2	0.4	0.1	0.11	2	0.032	0.034	0.84
15	9.3	0.5	0.1	0.13	1.8	0.036	0.04	0.81
17	13.4	0.7	0.12	0.15	2	0.049	0.049	0.86
18	15.8	0.8	0.13	0.16	2.2	0.047	0.054	0.91

Supplementary Table 19 Comparison of the communication costs and setup and online runtimes of SPIKE for the three networking configurations A: LAN + $10\,\mathrm{Gb/s}$, B: LAN + $1\,\mathrm{Gb/s}$, C: WAN and for cycle length $L = 3$. This table contains the individual costs of Phases 3 and 4 (Cycle and Solution Evaluation).

Pairs	Comm. [MiB]		Setup Phase [s]			Online Phase [s]		
Pairs	Setup	Online	A	B	C	A	B	C
Phase 3: Cycle Evaluation								
3	0.3	0	0.0061	0.0068	0.1	0.022	0.022	0.42
5	3.4	0.2	0.06	0.071	0.6	0.089	0.1	0.53
7	17.9	0.6	0.2	0.21	2	0.22	0.27	0.71
9	49.1	1.2	0.54	0.56	4.8	0.53	0.63	1.1
11	172.4	2.6	1.8	2	16	1.7	1.7	2.2
13	1,005.9	9.1	11	12	89	9.1	9.1	9.6
15	1,773.8	12.8	19	21	160	16	16	16
17	4,213.3	26.5	47	50	370	38	38	39
18	6,735.8	42	79	82	590	65	65	65
Phase 4: Solution Evaluation								
3	0	0	0.0024	0.0022	0.011	0.0038	0.0059	0.22
5	0.1	0	0.0083	0.0082	0.32	0.014	0.014	0.75
7	0.5	0.1	0.016	0.017	0.54	0.039	0.04	1.9
9	0.8	0.1	0.024	0.025	0.64	0.057	0.056	2.6
11	5.5	0.4	0.078	0.087	1	0.19	0.19	5.5
13	202.7	6.9	0.81	1.4	14	2.4	2.5	22
15	300	9.5	1.1	1.8	18	3.6	3.7	27
17	1,200.1	30.9	4.1	6	64	15	16	53
18	2,783.8	64.8	9.2	13	150	36	36	87

Supplementary Table 20 Comparison of the setup and online runtimes of SPIKE for the reduced medical factor compatibility matching and the full set in the two main networking configurations A: LAN + $10\,\mathrm{Gb/s}$, C: WAN.

Pairs	Comm. [MiB]		Setup Phase [s]		Online Phase [s]	
Pairs	Setup	Online	A	C	A	C
Reduced Medical Factor Set						
2	0.1	0	0.0084	0.34	0.045	3
50	14.9	0.3	0.14	1.7	0.26	3.4
100	59.8	1.1	0.29	4.4	0.81	4.4
150	134.7	2.5	0.55	8.5	1.9	5.8
200	239.5	4.4	0.91	15	3.8	7.7
250	374.4	6.9	1.4	23	6.4	11
300	539.2	9.9	2	31	9.4	14
350	734	13.4	2.5	41	14	20
400	958.8	17.5	3.2	53	18	26
450	1,213.6	22.1	4.2	65	25	32
500	1,498.3	27.3	5.3	80	31	37
550	1,813.1	33	6.3	96	38	48
600	2,157.8	39.3	7.2	110	45	56
650	2,532.5	46.1	9	130	53	64
Full Medical Factor Set						
2	0.1	0	0.013	0.88	0.047	3.4
50	44	11.8	0.51	4.6	1	5.2
100	177.1	47.1	1.3	14	4.7	12
150	399.2	105.9	2.8	29	12	24
200	710.5	188.3	5.1	48	22	41
250	1,110.9	294.3	7.6	71	35	64
300	1,600.4	423.8	12	100	51	92
350	2,179.1	576.8	14	140	66	120
400	2,846.8	753.4	18	180	86	160
450	3,603.7	953.5	23	230	110	200
500	4,449.6	1,177.2	28	280	140	250
550	5,384.7	1,424.4	35	340	170	300
600	6,408.9	1,695.2	41	410	200	350
650	7,522.2	1,989.5	48	480	240	420
